# Bacterial degradation of a plant toxin and nutrient competition with commensals trade off to constrain pathogen growth

**DOI:** 10.1128/msystems.00064-26

**Published:** 2026-06-26

**Authors:** Marco Mauri, Kerstin Unger, Jonathan Gershenzon, Rosalind J. Allen, Matthew T. Agler

**Affiliations:** 1 Theoretical Microbial Ecology, Institute of Microbiology, Faculty of Biological Sciences, Friedrich Schiller University Jena 163335, Jena, Germany; 2 Cluster of Excellence Balance of the Microverse, Friedrich Schiller University Jena 9378, Jena, Germany; 3 Plant Microbiosis Group, Institute of Microbiology, Friedrich Schiller University Jena 163335, Jena, Germany; 4 Department of Biochemistry, Max-Planck-Institute for Chemical Ecology 28298 , Jena, Germany; DOE Joint Genome Institute, Berkeley, California, USA

**Keywords:** virulence factors, microbiome, plant pathogens, public good, commensals, isothiocyanate, detoxification, biocontrol

## Abstract

Healthy plant leaves potentially host both commensal bacteria and opportunistic pathogens, which, under some circumstances, may cause disease. The interactions between commensals and opportunistic pathogens are generally poorly understood, but such understanding is crucial for developing effective biocontrol strategies. In *Arabidopsis thaliana*, isothiocyanates (ITCs) are defense metabolites that suppress most bacteria; commensals are especially affected as they do not express ITC resistance genes. The ITC hydrolase SaxA detoxifies ITCs, making it an important virulence factor for bacterial and fungal pathogens. To investigate pathogen-commensal interactions based on SaxA-mediated ITC degradation, we used five ITC-sensitive bacterial commensals and the opportunistic pathogen *Pseudomonas viridiflava* 3D9 (PS). All strains were isolated from healthy *A. thaliana* leaves. PS degrades 4-methylsulfinylbutyl-ITC (4MSOB-ITC) with SaxA. We examined commensal growth in the presence of 4MSOB-ITC, both in monoculture and in coculture with PS or a *saxA*-deficient mutant (PSKO). We used the growth data to develop a generalizable consumer-resource mathematical model incorporating ITC toxicity, ITC degradation, and nutrient use. We predicted and confirmed experimentally that the extent to which SaxA benefits the pathogen depends on its effects on commensals. In some contexts, commensal rescue and the resultant nutrient competition limit pathogen growth. In addition, we tested *in silico* how commensal ITC susceptibility, pathogen ITC degradation rates, and growth parameters affect the trade-off between SaxA-mediated virulence (strong pathogen growth) and commensal rescue (commensal growth). Our findings suggest that the effects of microbial traits—traditionally viewed as either virulence or plant-beneficial factors—are constrained in the microbiome context. This underscores the need to reconsider how such traits are classified in the context of plant-microbiome interactions.

**IMPORTANCE:**

Healthy plant leaves host a variety of bacteria; these can be beneficial, but some (opportunistic pathogens) can also be harmful under certain conditions. To design effective biocontrol strategies to sustainably protect plants, it is important to understand how opportunistic pathogens thrive as part of a healthy leaf microbiome. Plant defense metabolites, such as isothiocyanates (ITCs), which kill commensal leaf bacteria, and bacterial ITC resistance mechanisms, such as the ITC hydrolase SaxA, which are often expressed in pathogens and degrade ITCs, may play key roles in the plant microbiome composition. In this study, we explore how SaxA-mediated ITC degradation by a pathogen also benefits diverse ITC-sensitive commensals and how this, in turn, could shape microbiome stability and plant health. Using mathematical modeling based on growth data from *Pseudomonas viridiflava* with diverse commensals, we find that interaction dynamics can be explained by ITC detoxification and nutrient competition. We predict and experimentally confirm that conditions exist under which SaxA favors commensal growth so strongly that the pathogen is outcompeted for resources, thus not benefiting from its own virulence factor. Our findings suggest that the effects of microbial traits, including virulence factors, are context-dependent, especially when functioning as a public good in a community context like SaxA. Moreover, we propose that this concept, which has been known from antibiotic-degrading microbes, may be worth considering as well when studying plant-pathogen interactions under natural conditions where the commensal microbiome might play an important role in plant disease outcomes.

## INTRODUCTION

Pathogenic microbes have traditionally been defined based on Koch’s postulates, which identify a specific microbe as the causal agent of a specific disease; the disease-causing microbe is postulated to be present in sick but not in healthy individuals. In this framework, disease is presumed only to depend on a pathogen’s genetic capacity to produce virulence factors and evade host defenses to proliferate in a host (1). However, this view neglects how non-host or environmental factors can influence disease. In plant pathology, the so-called disease triangle rationalizes this context dependence, postulating that disease is not solely dependent on the presence of a pathogen but rather results from the interplay between plant host traits, opportunistic pathogens, and the environment. The concept has recently been extended to include interactions between pathogens and the resident plant microbiome, underlining the importance of co-colonizing microbes (2). In this context, commensal microbes can keep pathogens in check, for example, by outcompeting them, supporting plant health, and, in cases of disease, a pathogen may overcome this competition. This development in the concepts of health and disease reflects a shift in research in plant pathology from the one-pathogen-one-disease model toward understanding microbial and plant traits that help sustain a balanced microbiome and promote plant health.

An important plant trait that shapes microbial colonization of roots and leaves is the presence of antimicrobial plant specialized metabolites like isothiocyanates (ITCs), which suppress the growth of non-resistant microbes, often commensals (3). Microbial detoxification of antimicrobial metabolites is therefore a key process that contributes to the virulence of plant pathogens (4–7) and may also protect commensals. An important function of the commensal plant microbiome is to suppress plant pathogens by competing with them for nutrients (8, 9). However, the interplay between the production of plant defense metabolites, detoxification of these metabolites, and nutrient competition between pathogens and commensals is still poorly understood, especially in the context of complex plant microbiomes. In the context of the human gut, the degradation of antimicrobial drugs, such as antibiotics, can protect co-colonizing antibiotic-susceptible bacteria (10). Here, we propose that the effects of degradation of antimicrobial plant defense metabolites on the plant microbiome may be similar.

The plant microbiome, specifically the microbiome of healthy leaves, consists mainly of Proteobacteria, Actinobacteria, and Bacteroidetes (11, 12). These include both non-pathogenic commensal taxa that are often sensitive to plant defense metabolites and opportunistic pathogens, some of which can degrade plant defense metabolites. The presence of opportunistic pathogens even in healthy leaf microbiomes (13) raises questions about whether interactions with commensals might cause pathogens to shift from harmless leaf colonizers to disease-causing agents.

In the model plant *Arabidopsis thaliana,* aliphatic glucosinolates (GLS) are major leaf defense metabolites that are converted into antimicrobial ITCs when plant cells are damaged: the so-called “mustard-oil bomb” (14). The release of ITCs can be caused, for example, by insect herbivory (15) or by disruption of plant cells by microbial pathogens (16). ITCs can damage bacterial cells, for example, by impairing cell wall integrity (17), and our previous work showed that 4-methylbutylsulfinyl ITC (4MSOB-ITC) reduces the growth of plant-derived commensals (3). To overcome ITCs, both bacterial and fungal pathogens express virulence factors encoded by *sax* genes (survival in *Arabidopsis* extracts) that confer ITC resistance. Proteins encoded by *sax* genes include the efflux pumps SaxF, SaxG, and SaxD, as well as the ITC hydrolase SaxA (18). SaxA hydrolyzes various ITCs into their corresponding amines, rendering them non-toxic (19) and promoting virulence in various pathosystems (5, 18, 20).

Bacteria that do not have a specialized ITC hydrolase or efflux pumps, including commensal leaf colonizers and non-adapted pathogens, have varying levels of ITC susceptibility ranging from highly susceptible to almost resistant (3, 18). These mechanisms differ in their potential for collective effects; while efflux pumps are not generally expected to have significant effects beyond the carrying microbe (although efflux-mediated interactions are possible [21]), ITC detoxification by SaxA is expected to function as a public good that can influence the co-colonizing microbial community. We previously observed that ITC-sensitive commensal bacteria can benefit from SaxA-mediated ITC degradation (22). These bacteria can face very sudden exposure to ITC defense metabolites when the plant host comes under attack, and they probably also need to cope with low constitutive levels of ITCs arising from leaky transport of GLS (23), constant cycling between GLS and ITCs in intact plant tissues (24), and/or release of ITCs by GLS-utilizing bacteria (3). However, it is still unclear how SaxA-mediated ITC degradation affects commensals and pathogens in the context of the complex leaf community.

Here, we hypothesize that SaxA-mediated ITC degradation by the opportunistic plant pathogen *Pseudomonas viridiflava* not only increases its virulence but also acts as a public good to protect ITC-sensitive commensals, creating a trade-off in pathogenicity by enhancing competition of the pathogen with the rescued commensals for limited nutrients. To investigate this proposition, we used the ITC-degrading opportunistic pathogen *Pseudomonas viridiflava* 3D9 (PS) and five commensals that are sensitive to 4MSOB-ITC, a major GLS-derived defense metabolite in the widely used reference genotype *A. thaliana* Col-0 (18). All strains were previously isolated from healthy leaves of wild *A. thaliana* plants*,* making this a good model system for taxa that are likely to co-occur and interact in natural systems. We built a mathematical model based on our experimental measurements of microbial interactions mediated by the degradation of 4MSOB-ITC. We then used the model to predict when a trade-off occurs that is likely to be unfavorable to the pathogen but benefits the commensals, and we experimentally tested these predictions. This work reveals that the benefit of the “virulence factor” SaxA for pathogen growth is highly dependent on pathogen and commensal traits and the ITC concentration supplied by the plant.

## MATERIALS AND METHODS

### Bacterial strains used in this study

Commensal bacterial strains used in this study were *Stenotrophomonas* sp. SrG (hereafter: E), *Plantibacter* sp. 2H11-2 (hereafter: G), *Janthinobacterium* J4 (hereafter: K), *Brevundimonas* sp. 7B5 (hereafter: M), and *Rhodococcus* sp. 6G8 (hereafter: R), and the opportunistic pathogen *Pseudomonas viridiflava* 3D9 (hereafter: PS), which were isolated from healthy leaves of different *A. thaliana* genotypes (details in Table S1). A *saxA*-knockout mutant in the PS background (hereafter: PSKO) was generated in a previous study (22). All bacteria were grown on R2A agar plates or in R2A broth (yeast extract 0.5 g/L, peptone 0.5 g/L, casein hydrolysate 0.5 g/L, glucose 0.5 g/L, soluble starch 0.5 g/L, K_2_HPO_4_ 0.3 g/L, MgSO_4_ 0.024 g/L, sodium pyruvate 0.3 g/L, for plates: 15 g agar/L; pH = 7.2 ± 0.2 [25]) at 28°C, unless stated otherwise.

### Fluorescent tagging of PS and PSKO with mScarlet-I

Wild-type PS and PSKO were fluorescently labeled according to Schlechter et al. (26). Briefly, the pMRE-Tn7-145 plasmid containing the gene for the red fluorescent protein mScarlet-I, as well as chloramphenicol and gentamicin resistance cassettes, was conjugated from *E. coli* ST18 into both PS and PSKO strains. The transposon of the plasmid was integrated into the genome by a Tn-7 transposase, and bacterial cells that did not integrate the plasmid were counter-selected by incubation at 35°C to prevent replication of the temperature-sensitive plasmid. Absence of the delivery plasmid and the fluorescent protein, and absence of *E. coli* contamination, were checked via PCR as described in reference 26. The constitutive expression of mScarlet-I in colonies on agar plates and in liquid cultures was confirmed via fluorescence microscopy. Expression of the fluorescent protein did not significantly influence the overall growth of either strain at any 4MSOB-ITC concentration (Fig. S1). The labeled strains were therefore used throughout the experiments.

### Growth curves of mono- and cocultures of bacteria

All strains were streaked from glycerol stocks on R2A agar and incubated at 28°C. Individual colonies were used to inoculate R2A broth and shaken overnight at 28°C at 200 rpm. mScarlet-I-tagged PS and PSKO strains were pre-grown with appropriate antibiotics. The next day, the cultures were normalized to OD_600_ = 0.2 (monocultures) or 0.4 (cocultures). For pairwise cocultures, equal volumes of the commensal and PS or PSKO were combined, diluting each strain to a final OD_600_ = 0.2. For pairwise cocultures with different PS(KO):commensal ratios, the total OD_600_ was kept at 0.4, and the OD_600_ of PS(KO) and a commensal partner were mixed in different ratios [PS(KO):commensal = 100:1, 10:1, 1:1, 1:10, 1:100]. In a 96-well plate, three replicates per 4MSOB-ITC concentration were inoculated with 10 µL normalized culture in a total volume of 100 µL supplemented with 4MSOB-ITC ranging from 0 (pure DMSO) to 60 µg/mL. Negative growth controls (blanks) were inoculated with sterile R2A broth instead of bacteria. The plate was incubated at 28°C in a TECAN Infinite M Plex plate reader (i-control 2.0 software), and the OD_600_ and red fluorescence (excitation: 540 nm, emission: 595 nm, gain: 120) were measured every 30 min after 1 min of orbital shaking.

### Test of SaxA public good effect in a commensal bacterial community

Overnight cultures of all five commensals, PS, and PSKO (both tagged with mScarlet-I) were normalized to OD_600_ = 0.6 and combined in equal amounts to form synthetic communities (SynCom): all five commensals together (SynCom5), SynCom5+PS (hereafter: SynCom5+SaxA), and SynCom5+PSKO (hereafter: SynCom5−SaxA). Instead of PS or PSKO, sterile R2A broth was added to dilute SynCom5 such that in all SynComs each strain was present at an abundance corresponding to an OD_600_ of 0.1. A volume of 1,350 µL R2A was inoculated with 150 µL of the SynCom of interest in a 5 mL tube supplemented with a final concentration of 60 µg/mL 4MSOB-ITC, with five replicates per SynCom. The cultures were incubated on the shaker at 150 rpm at 28°C for 24 h. After 0, 3, 5, 7, 9, 11, and 24 h, 150 µL of the cultures were sampled, transferred first to a 96-well plate to measure the OD_600_ and red fluorescence on the plate reader, and then they were frozen at −20°C. The OD_600_ of the non-inoculated medium was monitored throughout, and, as expected, there was no increase in OD_600_ over time, so samples for DNA extraction (as sterility control) were only taken after 24 h. Samples for 4MSOB-ITC and 4MSOB-amine quantification were taken after 24 h as well, to confirm the degradation of the ITC in SynCom+SaxA. To enable absolute quantification of bacterial growth, before the DNA extraction, 2 µL of internal standard (ZymoBiomics, Spike-In Control, High Bacterial Loads, Zymo Research, Freiburg, Germany), which contains a defined cell number of the gram-negative bacterium *Imtechella halotolerans* and the gram-positive bacterium *Allobacillus halotolerans*, was added to each sample, except for samples from time point 24 h and the inocula, which received 8.5 µL. The DNA was extracted using bead beating and an SDS-buffer protocol, as described in reference 3.

The V3–V4 region of the 16S rRNA gene was amplified with primers 341F/799R (27), and amplicon sequencing libraries were prepared in a two-step PCR as described in reference 3. Pooled libraries were sequenced on an Illumina MiSeq instrument using a MiSeq Reagent Micro Kit v2 (300-cycles). The quality of raw reads was checked, and forward and reverse reads were combined using the dada2 package (28). Only reads with a quality score greater than 15 and no more than 1 expected error were included. ASVs were assigned using the SILVA 16S rRNA gene database (v 138.1) (29).

The data were analyzed and plotted using the phyloseq package (30) in R (version 4.4.2). The non-inoculated medium mainly amplified the added internal standard taxa, confirming both its sterility and the successful amplification of both the internal standard taxa (Fig. S2A). Positive controls (ZymoBIOMICS Microbial Community DNA Standard, Zymo Research, Freiburg, Germany) produced the expected results, and negative controls (nuclease-free water) had less than 250 reads and were not analyzed further (Fig. S2B). *Allobacillus halotolerans* was identified using all Bacillaceae reads, *Imtechella halotolerans* was identified using all Flavobacteriaceae reads, since neither family was part of the SynComs. Before further analysis, the addition of 8.5 instead of 2 µL of internal standard in the 24 h samples was corrected by dividing the reads of the two internal standard taxa by 4.25. Though both internal standard taxa were added in similar cell counts, the gram-positive *Allobacillus halotolerans* was underrepresented (Fig. S2A), suggesting lower efficiency of DNA extraction for gram-positive bacteria. Thus, gram-positive SynCom members (G, R) were normalized to Bacillaceae reads, and gram-negative members (PS, PSKO, E, K, M) were normalized to Flavobacteriaceae reads. The normalized results showed a clear increase in read counts over time in all three communities, reflecting growth (Fig. S2C). The increase in read counts was highest in SynCom5+SaxA and lowest in SynCom5, in line with the OD_600_ measurements (Fig. S3A). The resulting “absolute” abundances were plotted using the ggplot2 package in R (31).

### Quantification of 4MSOB-ITC and its degradation products via LC-MS

To quantify 4MSOB-ITC, 4MSOB-amine, and 4MSOB-ITC conjugated to glutathione (4MSOB-ITC-GSH) in supernatants of bacterial monocultures (*n* = 3) or 4MSOB-ITC and 4MSOB-amine in supernatants of the SynComs after 24 h (*n* = 5), we harvested 20 µL of the supernatant and diluted it in 180 µL MilliQ water. The diluted samples were frozen at −20°C until the analysis was carried out on an LC-MS as described (3). Briefly, external standard curves of 4MSOB-ITC (L-Sulforaphane, CAS 142825-10-3, Sigma Aldrich) and 4MSOB-amine (4-methanesulfinylbutan-1-amine, Enamine Germany GmbH, Frankfurt a.M., Germany) were measured together with the samples. 4MSOB-ITC-GSH was quantified relatively, and the data are shown in peak area units. 4MSOB-ITC, 4MSOB-amine, and 4MSOB-ITC-GSH were analyzed on an Agilent 1200 HPLC system (Agilent, Santa Clara, CA, United States) coupled to an API3200 tandem mass spectrometer (AB SCIEX, Darmstadt, Germany). All compounds were separated on an Agilent XDB-C18 column (5 cm × 4.6 mm, 1.8 μm, Agilent, Waldbronn, Germany). The mobile phase consisted of 0.05% (vol/vol) formic acid in ultrapure water as solvent A and acetonitrile as solvent B, at a flow rate of 1.1 mL/min. The elution gradient was 0–0.5 min, 3%–15% B; 0.5–2.5 min, 15%–85% B; 2.5–2.52 min, 85%–100% B; 2.25–3.5 min, 100% B; 3.5–3.51 min, 100%–3% B; 3.51–6 min, 3% B. The ion spray voltage was maintained at 5,500 eV in positive mode. The turbo gas temperature was set to 500°C, nebulizing gas to 60 psi, drying gas to 60 psi, curtain gas to 35 psi, and collision gas to 3 psi. Details of multiple reaction monitoring can be found in Table S2. Analyst Software 1.6 Build 3773 (AB SCIEX) was used to acquire and process the data.

### Model development

**Fig 1:**
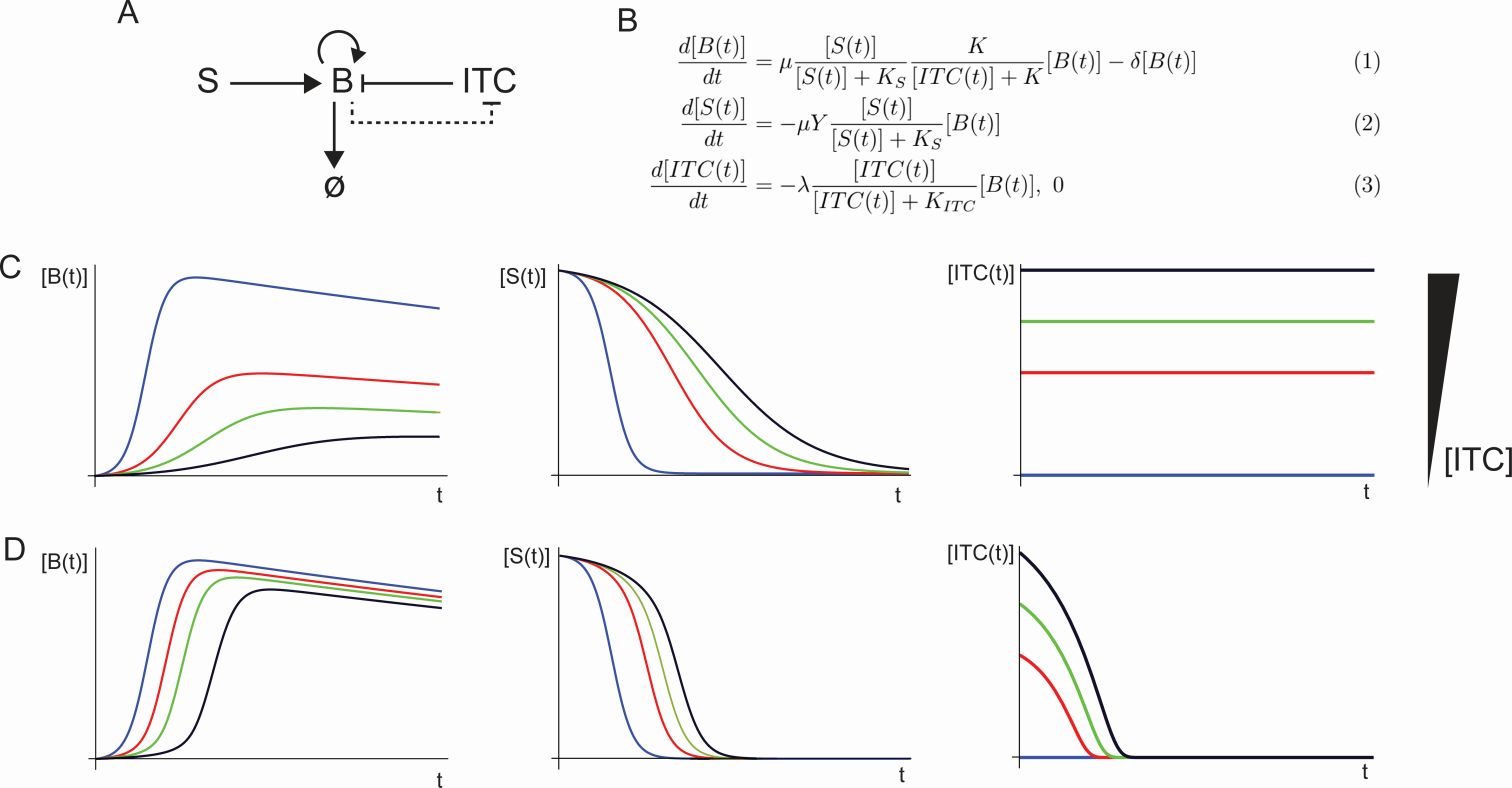
The mathematical model. (**A**) Schematic description of the model: the rate of change of bacterial biomass B is governed by a balance between growth due to consumption of the substrate S and death. ITC inhibits growth (and effectively also decreases the biomass yield; see Text S1), but ITC can be degraded by the degrader-strain PS (dotted line). (**B**) System of ordinary differential equations governing the growth dynamics of a given strain, nutrient consumption, and ITC inhibition and degradation, in the case of a single substrate. Biomass B increases by assimilating the substrate S via a Monod term with maximal growth rate μ and half-maximal concentration θ_S_, while ITC impairs growth via a repression term with half-maximal ITC concentration θ. Bacteria die with a constant death rate δ (equation 1). The substrate S is consumed via a corresponding Monod term and transformed into biomass; the constant Y converts between the units of biomass and substrate (equation 2). The degrader PS strain degrades ITC with maximal degradation rate λ and half-maximal ITC concentration θ_ITC_, while non-degrading strains are characterized by a null ITC degradation rate (equation 3). These equations correspond to a single substrate version of the model; the experimental data were fitted using a slightly more complex, two-nutrient version of the model for the PS and PSKO strains, see Text S2. (**C and D**) Qualitative illustration of the model dynamics for the biomass concentration [B(t)], substrate concentration [S(t)], and ITC concentration [ITC(t)] for the non-degrader strain PSKO (**C**) and degrader strain PS (**D**) for a range of values of the initial ITC concentration. For these simulations, we used the equations in panel **B** and parameter values in Table S3.

We developed a dynamical model based on ordinary differential equations to describe bacterial growth via nutrient assimilation, suppression of growth by the ITC, and degradation of the ITC by the opportunistic pathogen (Fig. 1A and B). In the absence of ITC, the ITC-degrading and non-degrading strains of PS are assumed to grow equally well, and nutrient utilization is modeled by decreasing nutrient concentration proportional to bacterial growth (blue curve, Fig. 1C and D).

However, increasing the ITC concentration affects the degrader and non-degrader strains differently. For the non-degrader strain (PSKO), ITC toxicity is modeled by slowing growth and decreasing the maximal biomass (i.e., the yield) at higher ITC concentrations (Fig. 1C). Since this strain does not degrade ITC, the ITC concentration remains constant in time (Fig. 1C). These model features are consistent with our experimental growth curves in which both the exponential growth rate and the maximal OD_600_ decrease with increasing ITC concentration.

The ITC degrader is modeled identically to the non-degrader (with the same ITC susceptibility), the only exception being that this strain degrades ITC with dynamics which were previously measured experimentally (32). Running the model for this strain, we observe that its growth is delayed by the presence of ITC, but it later achieves a growth rate and maximal biomass that are almost the same as in the absence of ITC, consistent with the fact that the ITC has been degraded (Fig. 1D).

The mathematical model in its simplest form is described by equations 1–3 (Fig. 1B). The biomass growth rate in equation 1 (Fig. 1B) depends on nutrient concentration via a standard Monod function (33) in which nutrient S is consumed with maximal growth rate μ and half-maximal concentration θ_S_. The Monod function is modulated by a term that describes inhibition by the ITC with half-maximal repression concentration θ (34). A smaller value of θ corresponds to greater ITC sensitivity. Bacteria are also assumed to die at a constant rate δ. To account for the observed decrease in maximal OD_600_ with increasing ITC, the rate of nutrient consumption (equation 2, Fig. 1B) depends on the nutrient concentration S via the same Monod function, multiplied by a yield coefficient Y (that converts units from nutrient to biomass concentration), but it does not depend explicitly on the ITC concentration. This is equivalent to having an effective growth yield that decreases with ITC concentration (Text S1). The ITC-degrading and non-degrading strains are modeled with the same equations and parameter values, except that the degrading strain degrades the ITC with maximal rate λ and half-maximal concentration θ_ITC_ (equation 3, Fig. 1B). The equations describing the non-degrader strains are also used to model commensals that are unable to degrade ITC, but with parameter values derived from their experimental measurements of ITC susceptibility and growth (Text S2). Within this model framework, the interaction between the pathogen and the commensal strains is mediated only by ITC degradation and competition for nutrients.

Our experimental growth curves for the ITC-degrader (PS) and non-degrader (PSKO) show an apparent change in growth dynamics (or “kink”) after 10–15 h that may correspond to utilization of a second nutrient within the complex R2A medium, i.e., a diauxic shift. To model this observed behavior, we extended the model to include a diauxic shift to a second nutrient (Text S2). This required three additional parameters describing the maximal growth on the second nutrient, a second Monod constant, and a diauxic repression coefficient (Text S2). This resulted in an excellent fit to the data (Fig. 4; Table S4).

The model results shown in Fig. 4 and 6 include this diauxic shift and therefore correspond to equations 4–7 and 8–12 of Text S2, for monocultures and cocultures, respectively.

### Fitting the model to experimental data to obtain parameter values

To obtain the parameter values µ, θ_S_, Y, θ, and δ that describe the growth curves of PS, PSKO, and the commensals, the mathematical model was fitted to monoculture growth curves, assuming that the biomass density B in our model is proportional to the experimentally measured OD_600_. To perform the fit, we wrote a custom routine in MATLAB that first subtracts background (media-only blank values, measured over time) from the raw growth curve data and then computes averages *S*_observed,*i*_ and standard deviations σ of the experimental OD_600_ measurements at each time point i over multiple replicate growth curves. The fit is then performed by minimizing the error-weighted chi-square function


$$\chi^{2}=\sum_{i}\frac{{\left(S_{\text{observed},i}-S_{\text{expected},i}\right)}^{2}}{\sigma_{i}^{2}},$$

where *S*_observed,*i*_ and *S*_expected,*i*_ are the OD_600_ values for a given time point *i*, as experimentally observed and as predicted by the model, respectively. The predictions for *S*_expected,*i*_ are obtained by numerical integration of the model equations for a test parameter set. The test parameter set is then systematically varied to find the parameter values that minimize the absolute difference between the model prediction and the experimental data, summed over all time points for that data set. To achieve this, we used the MATLAB routine fminsearch, and the system of ordinary differential equations was solved numerically via the function ode45. The standard deviations of the parameter values were computed using the procedure described in reference 35. All the parameter values in this study were therefore obtained by fitting experimental data for monocultures of either PS, PSKO, or commensals (see Fig. 4 and 5; Table S4).

### Model validation

We validated the model (which had been parameterized on monoculture data) by predicting growth curves (OD_600_ as a function of time) for 1:1 mixed cultures. We first verified the model by predicting the growth of a coculture containing equal amounts of fluorescently tagged PS(KO) and non-tagged PS(KO) at different ITC concentrations (Fig. S4A and B). In this case, where the two strains are described by the same equations (Text S2) and the system differs from the monoculture only in the initial conditions, the model predictions were in good agreement with the data. Then, we predicted growth curves for 1:1 mixed cultures of the degrader (PS) and non-degrader (PSKO) strains with different ITC concentrations. For mixed cultures, the model outcome depends on two effects: the two strains compete for nutrients, and PS degrades the ITC, protecting PSKO (see Text S2 for the equations corresponding to the mixed culture model). The model’s predictions were tested experimentally by measuring the dynamics of OD_600_ in mixed cultures of PS and PSKO. The model predictions for the OD_600_ of the mixed cultures were in good agreement with the experimental data (Fig. S4C and D). Moreover, the model also allows us to predict the dynamics of the PS and PSKO subpopulations within the mixed culture, revealing how PS promotes the growth of PSKO compared to the PSKO monoculture. Indeed, the model predicts that since SaxA functions as a public good, PS and PSKO grow equally well within the coculture (Fig. S4E and F); however, since nutrients are shared between the two subpopulations, competition suppresses the maximal population size of each strain compared to monoculture (Fig. S4D). The good agreement between our model predictions and measured growth curves suggests that the model, while conceptually simple, reproduces the essential biological mechanisms that are at play in our system, namely, protection of non-ITC-degraders by degraders, together with competition for nutrients.

## RESULTS

### Commensal leaf bacteria are sensitive to 4MSOB-ITC to different extents

**Fig 2:**
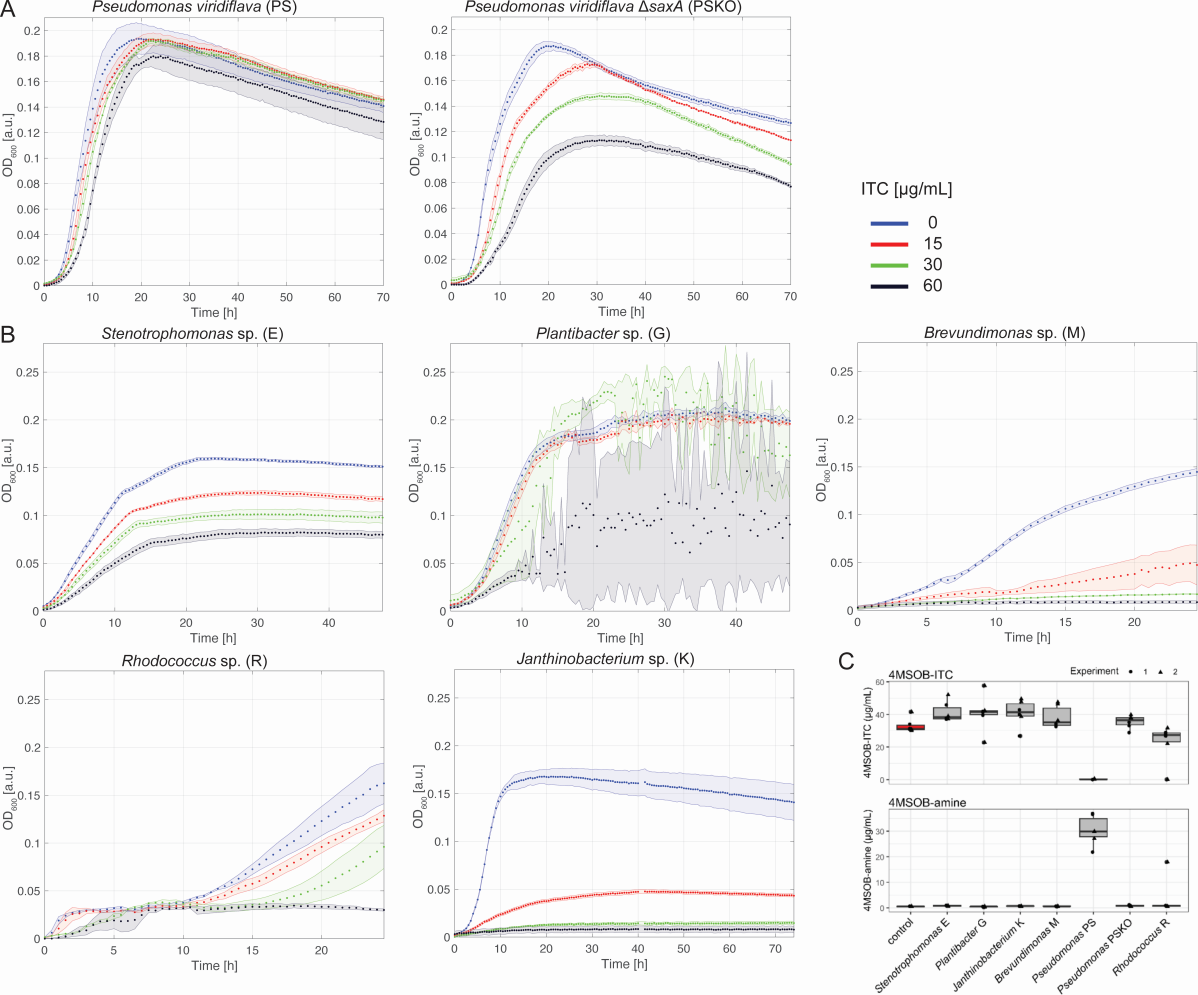
Leaf bacteria are sensitive to 4MSOB-ITC to different extents. Growth curves of PS and PSKO (**A**) and of the five selected commensals (**B**) in R2A broth supplemented with different 4MSOB-ITC concentrations at 28°C. Dots represent means, shaded areas depict standard deviations of one experiment with *n* = 3 technical replicates. With higher ITC concentrations, *Plantibacter* sp. G formed cell aggregates, resulting in high standard deviations. (**C**) Quantification of 4MSOB-ITC and its breakdown product 4MSOB-amine in the supernatant of monocultures of the five commensals, PS, and PSKO, after 16 h of incubation. The different symbols illustrate two independent experiments (*n* = 3 each). Boxplots summarize the data of both experiments. Red boxplots show controls that were not inoculated with bacteria, gray boxplots show data for the bacterial strains.

We screened a diverse set of commensal bacteria isolated from *A. thaliana* leaves (Table S1) to find strains that could benefit from SaxA-mediated ITC degradation. First, we checked for SaxA-like activity, although to the best of our knowledge, it has not previously been described in non-pathogenic bacteria. To our surprise, 4 out of 13 tested commensal taxa degraded 4MSOB-ITC to 4MSOB-amine, which is the typical degradation product of the ITC hydrolase SaxA (Fig. 2C; Fig. S5A). From the remaining nine taxa, we selected five (*Stenotrophomonas* sp. E, *Plantibacter* sp. G, *Janthinobacterium* sp. K, *Brevundimonas* sp. M, and *Rhodococcus* sp. R [Fig. 2B]) that are phylogenetically diverse and represent the major leaf colonizing phyla Alphaproteobacteria, Gammaproteobacteria, and Actinobacteria (Table S1). They all grew to detectable levels within 24–48 h in R2A medium and showed variable susceptibility to 4MSOB-ITC. Among the selected five commensals, *Stenotrophomonas* sp. E was the most ITC-resistant, while the least resistant strain was *Janthinobacterium* sp. K (Fig. 2B; Fig. S5B). To control for SaxA-independent interactions between the pathogen PS and the commensals, we used a *saxA* knockout mutant (PSKO). As expected, PSKO was more sensitive to high concentrations of the ITC than the wild-type PS (Fig. 2A and C). Previous quantification of the kinetics of 4MSOB-ITC degradation by PS has shown that the dose of ITC supplied is fully degraded in 3–6 h under the same culture conditions (32).

### SaxA-mediated rescue of diverse commensals in a community is dependent on their ITC sensitivity

**Fig 3:**
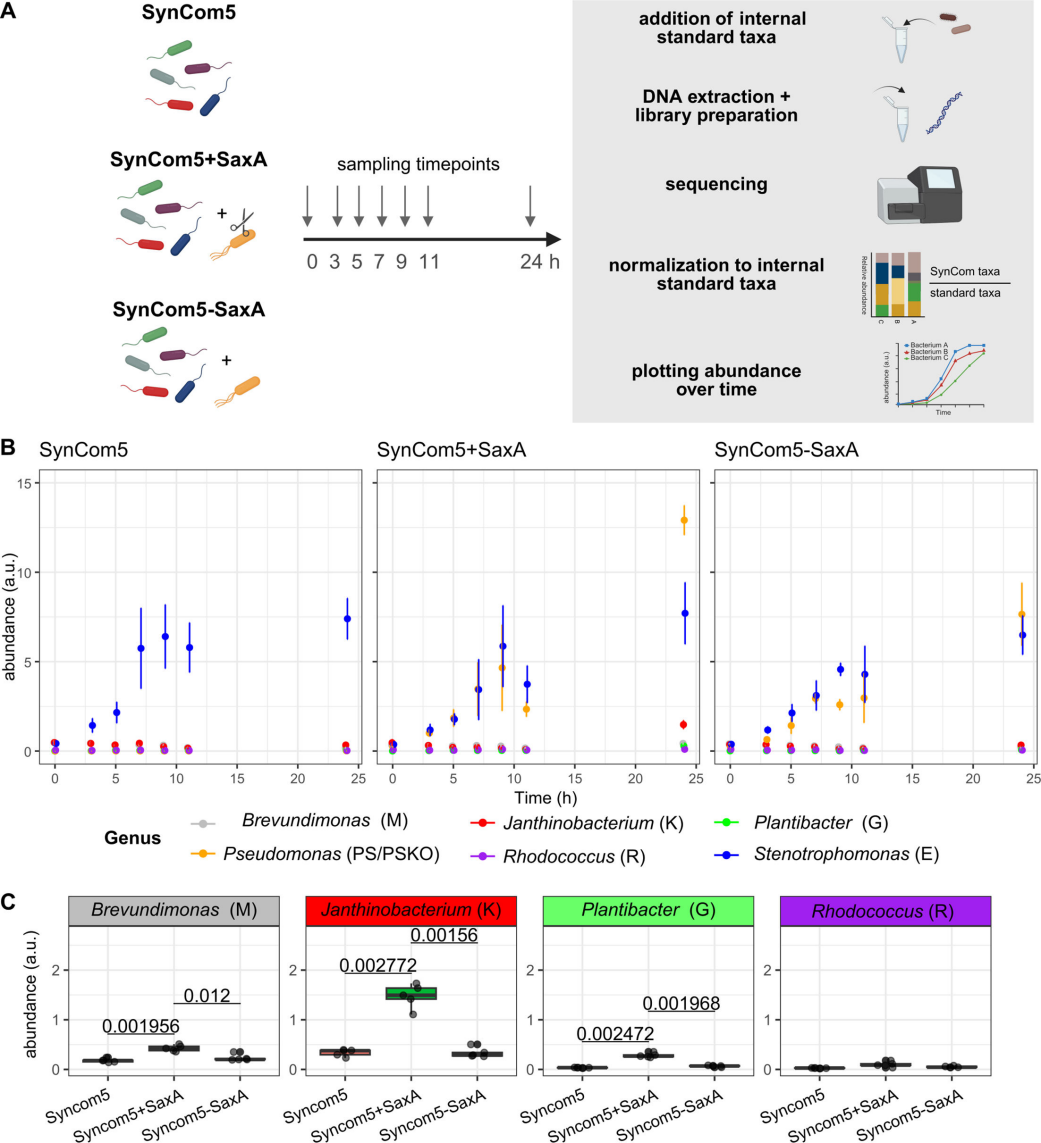
Growth of commensals in a synthetic community with or without SaxA. (**A**) Experimental design: all five commensals were mixed in equal amounts (SynCom5), and either PS (SynCom5+SaxA) or PSKO (SynCom5−SaxA) was added. The communities were inoculated in R2A broth supplemented with 60 µg/mL 4MSOB-ITC (*n* = 5 technical replicates) and sampled over 24 h. Internal standard taxa were added before DNA extraction and subsequent library preparation. After sequencing, the gram-positive SynCom taxa were normalized to the gram-positive standard taxa, and the same for the gram-negative ones. Created with Biorender.com. (**B**) Normalized absolute abundances (a.u. = artificial units) of each SynCom taxon over the course of 24 h. Each dot represents the mean of five replicates; standard deviations are depicted as whiskers. (**C**) Normalized absolute abundances (a.u. = artificial units) of low-abundance commensals after 24 h. Dots show individual samples (*n* = 5 technical replicates), boxes depict the median ± interquartile range, whiskers show ±1.5× interquartile range. Differences between treatments were assessed using *t*-tests; only significant comparisons (*P* < 0.05) are shown (adjusted *P* values, using the Bonferroni method).

Previously, we had shown that the commensal G benefits in an ITC-concentration-dependent manner from SaxA-mediated ITC degradation by PS (32). To assess the effect of SaxA within a community, we performed growth experiments with a synthetic community (SynCom) containing all five of our selected ITC-sensitive commensals (SynCom5), together with PS (SynCom5+SaxA) or with PSKO (SynCom5−SaxA) (Fig. 3A). The liquid medium was supplemented with a high 4MSOB-ITC concentration (60 µg/mL), which is sufficient to strongly inhibit the growth of most commensals (Fig. 2A). We took samples for 16S rRNA gene amplicon sequencing at regular intervals and at the end of the experiment. ITC quantification after 24 h confirmed its degradation only in SynCom5+SaxA (Fig. S3B). The absolute microbial abundance, as measured by both normalized amplicon sequencing data and OD_600_, was, as expected, highest for the SynCom5+SaxA community. However, perhaps surprisingly, SynCom5 without PS or PSKO also grew significantly at this high ITC concentration (Fig. S2A and S3A). The most ITC-resistant commensal strain E dominated all three communities, regardless of the presence of *Pseudomonas* or SaxA (Fig. 3B; Fig. S6). Commensals M, G, and especially K benefited from SaxA, showing some growth in SynCom+SaxA but not in SynCom−SaxA after 24 h; commensal R did not grow in the community setting within 24 h (Fig. 3C; Fig. S6). Thus, in the presence of high levels of ITC, SaxA is a public good that can simultaneously benefit diverse sensitive commensals.

### Commensal and pathogen growth can be quantitatively described by fitting model parameters to monoculture growth curves

**Fig 4:**
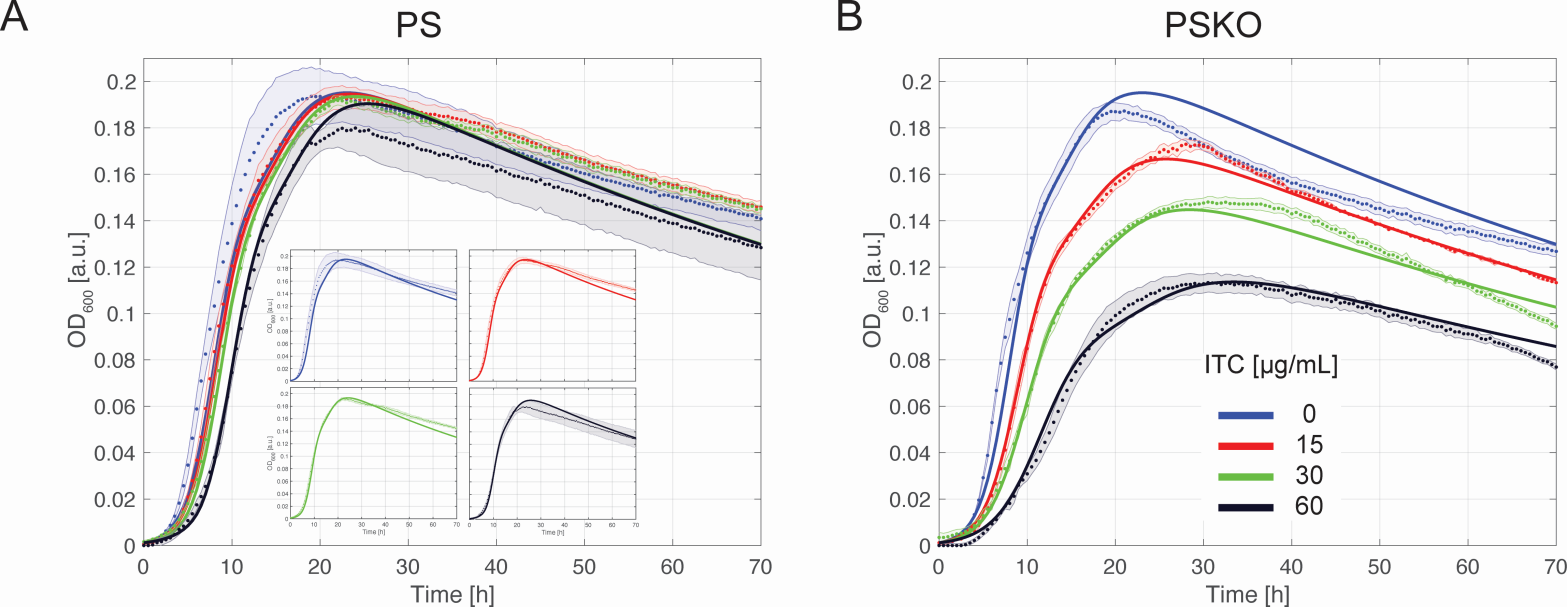
Fits of PS and PSKO monocultures. Fits of the mathematical model (including diauxic shift) to the experimental growth curves for PS (**A**) and PSKO (**B**) monocultures at increasing ITC concentrations. Experimental data points (dots) represent the mean of three technical replicates, with standard deviations indicated by shaded regions. Model predictions are depicted by solid lines. For clarity, the insets in panel A show the same fits for the PS monoculture at the different ITC concentrations, as separate plots.

To dissect the microbial interactions responsible for the observed public good function of SaxA in the SynCom, we developed a mathematical model, which we parameterized using monoculture growth data. The model consists of a set of ordinary differential equations describing the growth of each bacterial strain, inhibition by ITC, and degradation of ITC. It makes qualitatively distinct predictions for the growth dynamics of ITC-degrading and non-degrading strains in the presence of ITC (Fig. 1; see Materials and Methods). Model parameters for PS and PSKO were estimated by fitting to monoculture growth curves for a range of ITC concentrations (Fig. 4), taking into account a diauxic shift in nutrient usage on the complex R2A medium (see Materials and Methods, Fig. S7; Text S2); parameter values were assumed to be identical for PS and PSKO apart from the ITC degradation rate (λ in the model; see Fig. 1), which is zero for PSKO. The model produces an excellent fit to our monoculture data for PS and PSKO across the full range of ITC concentrations used in this study (Fig. 4; Table S4).

**Fig 5:**
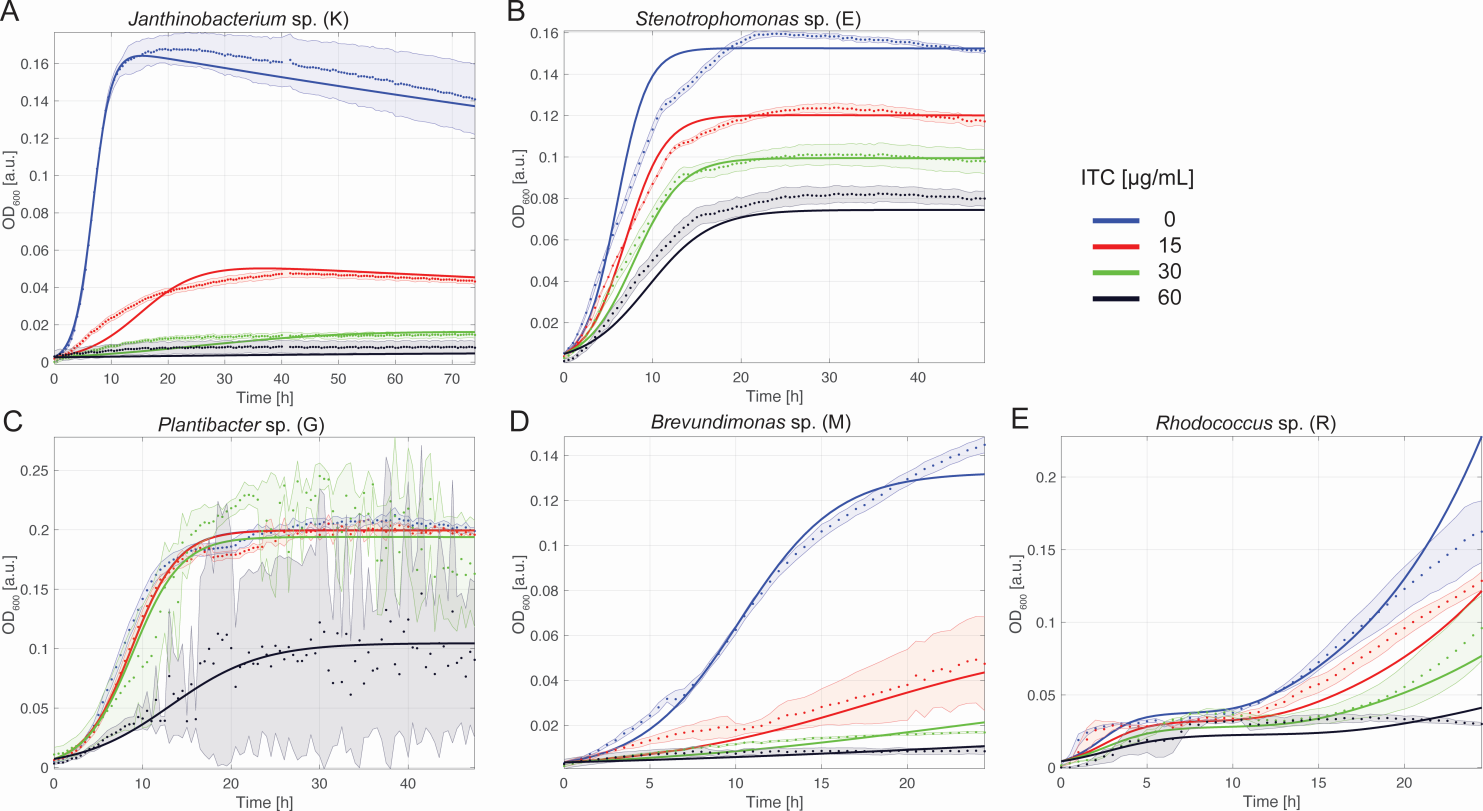
Model fits to monoculture growth data for the commensals. The single-nutrient model fitted to monoculture growth data for K (**A**), E (**B**), G (**C**), and M (**D**), but a model with diauxic shift fitted better for R (**E**). Parameter values resulting from these fits are given in Table S4. Different ITC concentrations are represented by distinct colors. The data points and shaded areas denote the mean and standard deviation of the experimental data with three technical replicates, while the solid lines represent the model fits.

We next used the same model, with some modifications, to describe the growth of each of the five commensal strains in monoculture, over a range of ITC concentrations. These strains did not degrade ITC in our experiments. The model parameters for each commensal strain were obtained by fitting the model without ITC degradation to the respective monoculture growth curves. We tested whether including a diauxic shift (i.e., a two-nutrient model, as we used for PS and PSKO) improved the fit, but this was the case only for R (Fig. 5E; Fig. S8). Therefore, we used a single-nutrient model for all the commensals. For strains K and G, the growth rate depended non-linearly on ITC concentration (Fig. 5A and C); therefore, we included a cooperative (Hill-type) inhibition function for these two commensals. During our experiments, we also noticed by visual inspection that G and R showed biofilm and aggregate formation in the microplate wells, especially at later time points and for higher ITC concentrations (Fig. 5C and E; Fig. S9). Since this likely interferes with the OD_600_ measurements, the ability of our model to predict the growth dynamics of these strains may be limited, especially for times later than 24 h, perhaps explaining the poorer fits in Fig. 5C and E. The full model used for each commensal strain, together with model fits and parameters, are described in Text S2 and Table S4. Thus, the simple model formulation of Fig. 1, with minor modifications, can describe monoculture growth of a range of commensals, with the exception of biofilm/aggregate formation. For the remainder of this study, we will focus on the most and the least ITC-sensitive strains, K and E, respectively (Fig. 5A and B).

### ITC detoxification and nutrient competition control the growth dynamics of pairwise cocultures

Next, we used the fitted model to predict SaxA-mediated interactions. Specifically, we predicted the growth of pairwise cocultures of commensals with either PS or PSKO at different ITC concentrations. The model accounts for nutrient competition (since a maximum population size emerges from the co-consumption of shared nutrients, as explicitly modeled in equations 10 and 11 of Text S2), as well as SaxA-mediated ITC degradation; by comparing model predictions to coculture data, we aimed to assess whether these two mechanisms could account for the observed coculture data.

**Fig 6:**
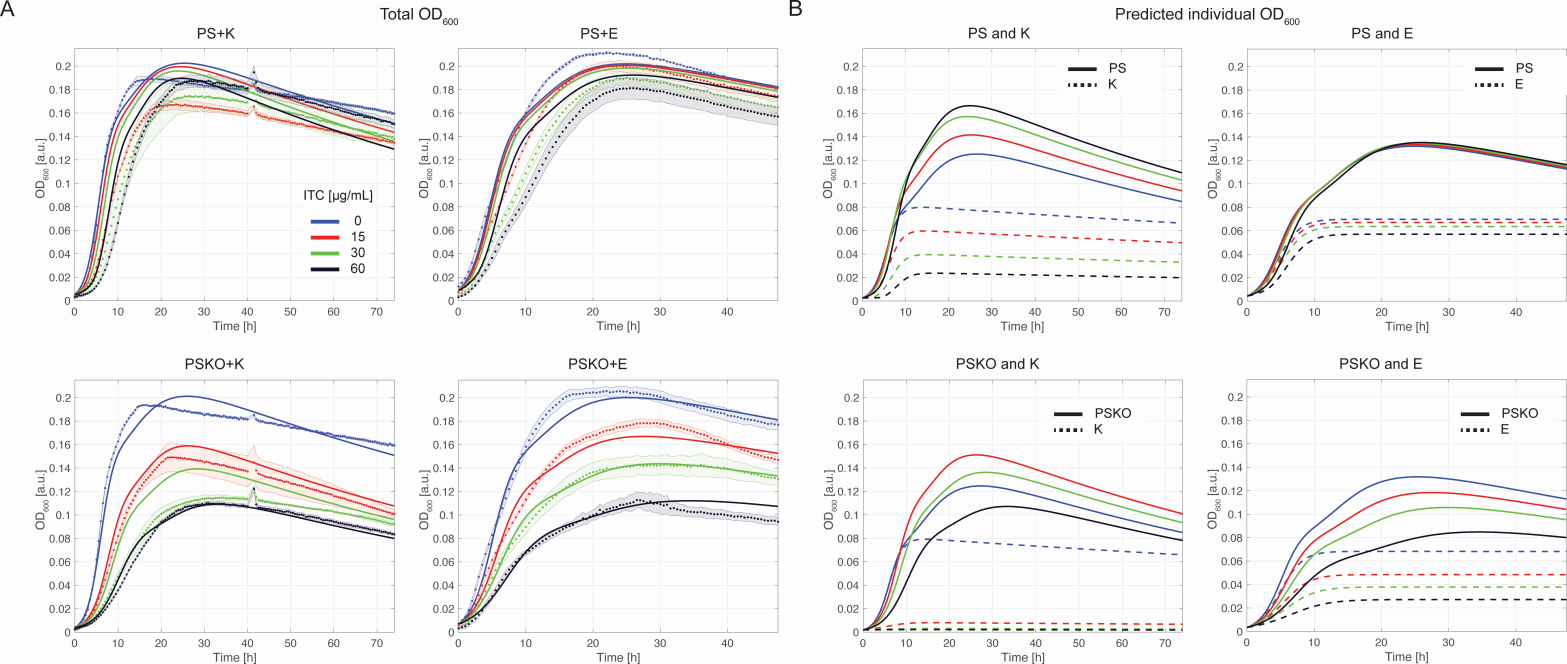
Model predictions for bacterial biomass in pairwise cocultures of PS or PSKO with commensal K or E. (**A**) The model prediction for the total biomass dynamics (OD_600_) of the pairwise coculture starting with the same amount of either PS or PSKO and the commensal for different ITC concentrations are shown by the solid lines and compared to the experimental data (dots, means of three technical replicates, standard deviations as shaded region). (**B**) Predicted separated growth dynamics of the biomass of either PS or PSKO and the commensal K or E in pairwise cocultures. Solid lines show the predicted OD_600_ of PS or PSKO, and dashed lines show the OD_600_ of the commensal strain.

Model predictions for the total OD_600_ of cocultures of PS/PSKO with either commensal K or E showed good agreement with our experimental data (Fig. 6A). The model also predicted that PS/PSKO is dominant, while the commensal contributes up to one-third of the total biomass (Fig. 6B). Comparing predictions for the most and least ITC-sensitive strains, the model shows that the more ITC-sensitive strain K benefits more from SaxA, whereas the more resistant strain E shows only a moderate increase in growth in the presence of PS versus PSKO, especially at low ITC levels. Despite the above-mentioned biofilm/aggregate formation by strains G and R (Fig. S9), the model predictions for both of these commensals in coculture with PS/PSKO were in good agreement with the data, probably because of the dominant contribution of PS/PSKO to the biomass (Fig. S10).

**Fig 7:**
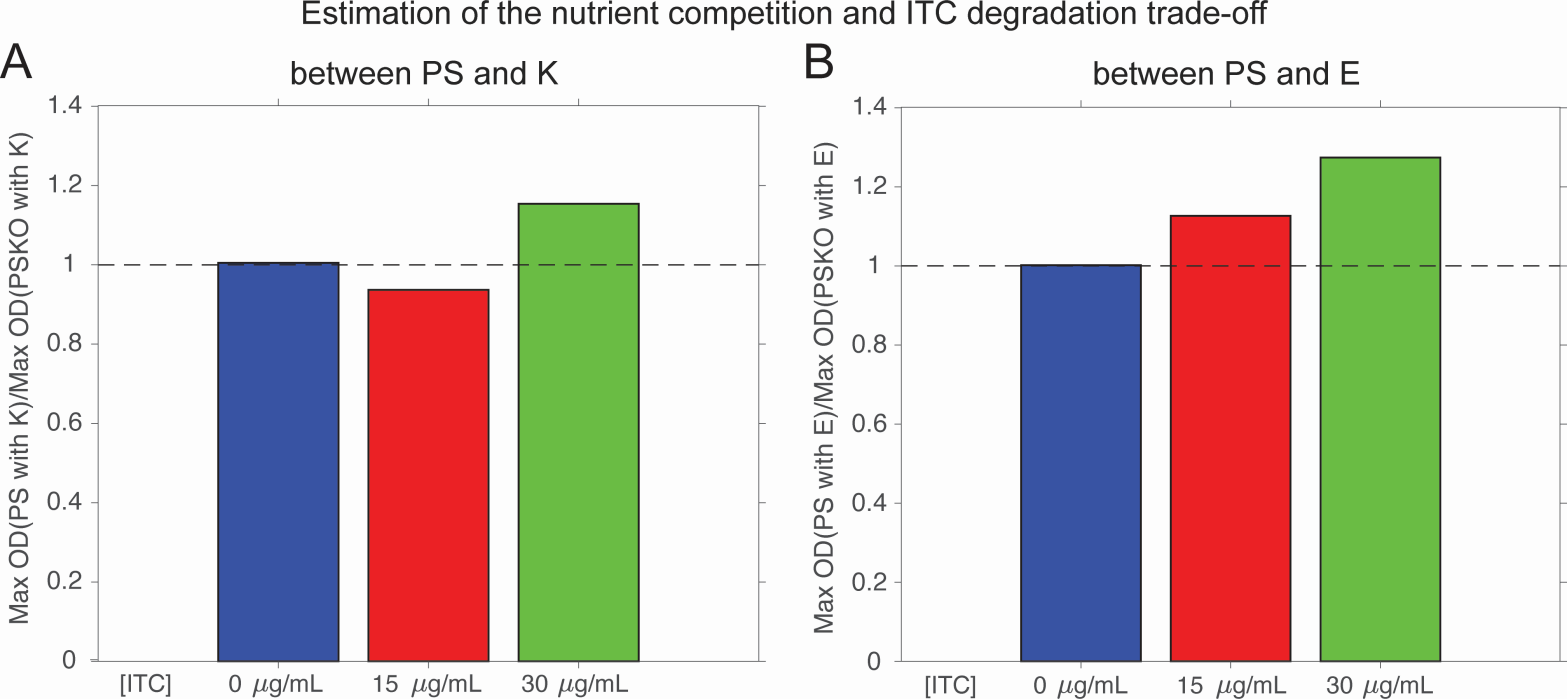
Quantification of the benefit for PS of SaxA-mediated commensal rescue. We computed the ratio of the maximal OD_600_ for PS to the maximal OD_600_ of PSKO, when either strain is in coculture with commensal K or E (panels **A and B,** respectively). The ratios are calculated from the maximal predicted OD_600_ values shown in Fig. 6B, for varying ITC concentrations (0, 15, and 30 µg/mL). A ratio below 1 indicates that PS experiences a growth penalty for rescuing the commensal, as the rescued population subsequently depletes shared nutrients. A ratio above 1 indicates that PS benefits from rescuing the commensal. (**A**) Data for the highly ITC-sensitive commensal K. PS experiences a penalty for rescuing the commensal at 15 µg/mL ITC but benefits from the rescue at 30 µg/mL. (**B**) Data for the ITC-tolerant commensal E. PS benefits from rescuing this commensal at both 15 and 30 µg/mL ITC.

Thus, our mathematical model that includes only nutrient competition and ITC inhibition and degradation successfully predicts the growth dynamics of pairwise cocultures of commensals together with either PS or PSKO, and pairwise cocultures. These results indicate that SaxA functions as a public good independently of the interaction partner, while also pointing to an important role for nutrient competition. In the model, nutrient depletion due to competition among community members limits the total population size. In coculture, nutrient resources are shared between the PS/PSKO and the commensal strain; consequently, the model predicts that a higher abundance of commensals (due to SaxA-mediated rescue) restricts PS/PSKO growth, and vice versa. This reciprocal limitation is indeed evident in our experimental data when comparing the maximal OD_600_ values of the curves representing PS and PSKO in Fig. 6B. At an intermediate ITC concentration (15 µg/mL), which only mildly decreases the OD_600_ of PSKO and has no measurable effect on the OD_600_ of PS monocultures (Fig. 4A and B), the maximal OD_600_ of PS is further reduced in the presence of the rescued commensal strain K, demonstrating competitive interactions under nutrient limitation. To directly visualize this trade-off, we computed the ratio of the maximal biomass, as measured by OD_600_, achieved by PS vs PSKO in cocultures with commensals K or E (Fig. 7). A ratio below 1 indicates that rescuing the commensal penalizes PS overall (relative to PSKO) through increased nutrient competition with the commensal, despite the direct benefit to PS growth from degradation of the ITC. A ratio above 1 indicates that ITC degradation is beneficial for PS, despite any increased nutrient competition due to pathogen rescue. As expected, in the absence of ITC, the ratio is 1 (i.e., PS and PSKO grow equally well in the cocultures). In the presence of ITC, we observe differing effects of ITC degradation on PS growth for the two commensals. For the sensitive commensal K, a clear trade-off appears at 15 µg/mL (Fig. 7A): PS rescues K, which then suppresses PS by competing for shared nutrients, resulting in a ratio lower than 1. At higher ITC concentration (30 µg/mL), however, rescue of the commensal is less effective, leading to reduced nutrient competition, so that ITC degradation is beneficial for PS (the ratio is greater than 1). In contrast, the other commensal, E, is much less sensitive to ITC and is able to grow in coculture with either PS or PSKO (i.e., it does not require rescue; Fig. 7B). Consequently, nutrient competition effects are similar for the cocultures with PS and PSKO, but PS benefits directly from clearing the ITC, leading to a ratio greater than 1. Taken together, this analysis shows that the competitive benefit or cost of providing this public good within the leaf community may be highly context-dependent.

### A rescue index and a pathogen suppression index illustrate the trade-offs involved in SaxA-mediated biocontrol of the opportunistic pathogen

**Fig 8:**
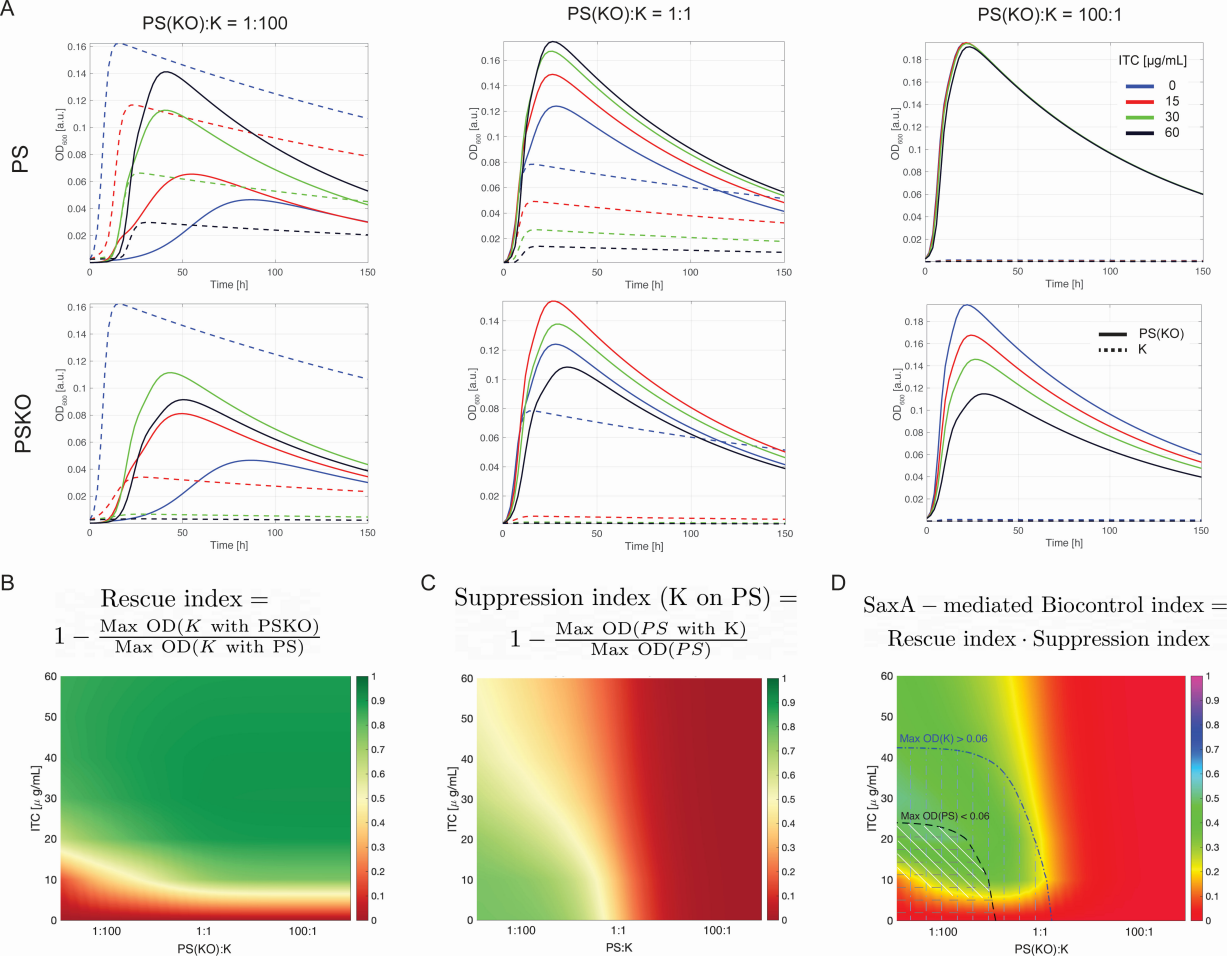
Three indexes to quantify the trade-off between commensal rescue and pathogen growth control. (**A**) Predicted growth dynamics of OD_600_ of PS and PSKO together with K at different initial ratios and ITC concentrations. Solid and dashed lines show the OD_600_ dynamics of the opportunistic pathogen and the commensal, respectively. (**B–D**) All three indices summarize the growth dynamics of PS(KO) and commensals at different ITC concentrations and PS(KO):commensal ratios. (**B**) The rescue index quantifies the extent to which SaxA rescues the growth of a commensal (in this case, K) from ITC inhibition, as detailed in Text S3. It compares the maximal OD_600_ (shown in heatmaps in Fig. S12) of the commensal strain with and without SaxA-mediated ITC degradation and ranges from 0 (no rescue) to 1 (complete rescue). (**C**) The suppression index quantifies the extent to which the growth of the commensal reduces the growth of PS. It compares the maximal OD_600_ of PS in the presence and absence of the commensal strain (in this case, K; see also the heatmaps in Fig. S12) and ranges from 0 (minimal suppression of PS) to 1 (maximal suppression of PS). (**D**) The SaxA-dependent biocontrol index combines the rescue index and the suppression index. The biocontrol index ranges from 0 (low K rescue and low PS suppression) to 1 (high K rescue and high PS suppression). This index is based on relative growth comparisons between PS(KO) and the commensal. To factor in the absolute bacterial abundances, we introduce abundance thresholds for effective biocontrol: the black dashed line and blue dot-dashed line indicate an absolute OD_600_ of 0.06 for PS (black) and K (blue). The white hatched region defines the region where SaxA-mediated biocontrol is maximal, the abundance of PS is below the threshold, and the abundance of K is above the threshold.

Up to now, we studied cocultures in which the opportunistic pathogen PS and the commensal strain were initially in equal abundance (Fig. 6B). However, in the context of plant leaf colonization, the abundance ratio between opportunistic pathogen and commensals may vary greatly: in a diseased plant, an opportunistic pathogen may outnumber other colonizers, while in a healthy plant, it may be outnumbered by commensals. Additionally, ITC concentration can vary over a wide range, from low levels in healthy tissue to high levels immediately after tissue damage. To understand the trade-off between ITC degradation by the opportunistic pathogen, which benefits commensals, and competition for resources between the opportunistic pathogen and the commensals, which may limit pathogen abundance, we used our model to predict how varying both the ITC concentration and the initial ratio of abundances of pathogen and commensal could influence the coculture dynamics of PS/PSKO with the ITC-sensitive commensal K. Our model predicts how the effects of ITC degradation and nutrient competition combine to shape pathogen-commensal cocultures. These effects are strongly context-dependent: for example, at 15 µg/mL ITC, the outcome for the commensal K depends on the initial mixing ratio (red lines, Fig. 8A; Fig. S11). If PS is at least as abundant as K (1:1 or 100:1), PS monopolizes nutrients and suppresses K. If PS is much rarer (1:100), it still degrades ITC, allowing K to grow, which in turn restricts PS through nutrient competition (Fig. 8A; Fig. S11).

To characterize the complex role of SaxA-mediated ITC degradation across different conditions, we defined three indices to summarize the key features of the model predictions.

First, we defined a “rescue index” that quantifies the extent to which the commensal is rescued by SaxA from ITC inhibition (Fig. 8B, detailed description in Text S3), ranging from 0 (minimal rescue) to 1 (maximal rescue). As expected, the rescue index is larger at high ITC concentrations, where SaxA-mediated ITC degradation is more relevant (Fig. 8B) but is almost independent of the mixing ratio PS:K, since even a small abundance of PS is sufficient to fully rescue the sensitive commensal.

Next, we summarized the suppression of PS growth via nutrient competition with the commensal by defining a “suppression index” that quantifies the extent to which the growth of the commensal reduces PS growth (Fig. 8C, more detailed description in Text S3); this is a number ranging from 0 (minimal suppression of PS) to 1 (maximal suppression of PS). The suppression index depends strongly on the initial PS:K ratio. When PS is initially present in excess (PS:K = 100:1), the commensal is not able to suppress the fast-growing opportunistic pathogen (Fig. 8C, red region, suppression index is low). However, if the initial ratio of PS is low and the ITC concentration is also low, nutrient competition becomes significant, and PS is suppressed by K (Fig. 8C, green region, suppression index is high). Interestingly, the suppression index and the rescue index show qualitatively different context-dependence: maximal rescue of the commensal by PS occurs when the ITC concentration is high and PS:K is low (Fig. 8B); these conditions are associated with intermediate values of the suppression index, since the growth of PS is only partially suppressed by nutrient competition with K (Fig. 8C).

To capture the trade-off between SaxA-mediated rescue of the commensal and pathogen suppression via nutrient competition, we also defined a “SaxA-mediated biocontrol index” (Fig. 8C, more detailed description in Text S3) that expresses the combined effect of the rescue and the suppression indexes. The SaxA-mediated biocontrol is maximal for low initial PS abundance and high ITC, depicted by the green region in Fig. 8D, corresponding to conditions where both SaxA-mediated rescue and pathogen suppression are high (i.e., the green regions of Fig. 8B and C). Under these conditions, SaxA-mediated ITC degradation by PS is predicted to rescue commensal growth while, at the same time, PS growth is limited by commensals.

So far, our analysis has focused on relative differences in pathogen and commensal growth under different conditions. However, for plant health, the absolute numbers of pathogens and commensals matter. Therefore, we further refined the predicted regions of SaxA-mediated biocontrol by applying threshold criteria. We suppose that, for effective biocontrol, the absolute abundance of the pathogen must remain below a threshold value, while the absolute abundance of the commensal must remain above a threshold. The relevant values of these thresholds should be determined in future work; here, for illustration, we arbitrarily set them to OD_600_ = 0.06, producing the blue and black lines in Fig. 8D. The white hatched region indicates the predicted region of effective biocontrol where the biocontrol index is high, while PS abundance remains below the threshold and the abundance of the commensal K is above the threshold (see also heatmaps in Fig. S12; Text S3).

### SaxA-mediated negative effect on PS growth is confirmed by experimental data for PS and K cocultures

**Fig 9:**
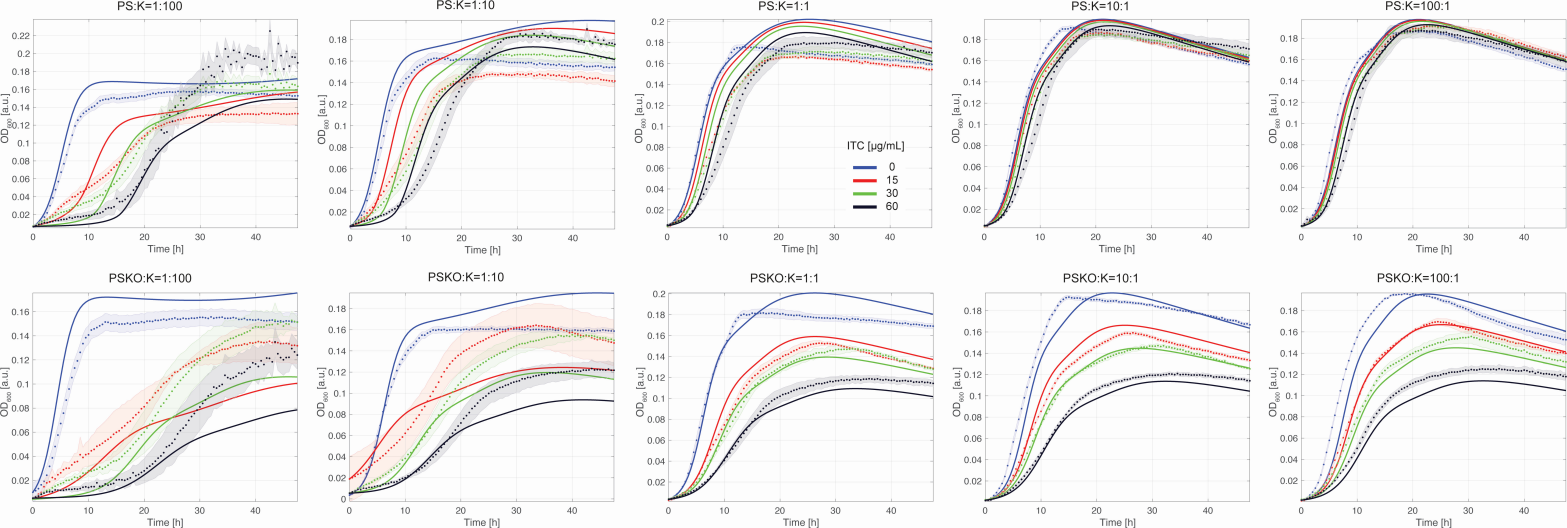
Comparison between model prediction and experimental data of PS(KO):K at different initial ratios and ITC concentrations. Model predictions (solid lines) and experimental measurements (average as dots and shaded regions as standard deviations of three independent replicates) for the OD_600_ of the pairwise cultures PS:K and PSKO:K, in the first and second row, respectively, and for different PS(KO):K ratios (1:100, 1:10, 1:1, 10:1, 100:1) at a range of ITC concentrations.

We tested the model predictions by measuring the growth dynamics of PS(KO):K cocultures at different mixing ratios (1:1, 1:10, 10:1, 1:100, and 100:1). We found excellent agreement between the model predictions and the measurements over the full range of ITC concentrations (Fig. 9), suggesting that nutrient competition and ITC degradation are indeed the key drivers of coculture dynamics.

Our model predicted that the virulence factor SaxA would not just benefit the pathogen PS but at high ITC concentrations and high abundance of K, the ITC-sensitive commensal would indirectly benefit as well (corresponding to high values of the rescue index; Fig. 7B). This effect emerges clearly in our experimental data when comparing the total OD_600_ (growth of both strains together, Fig. S13A) with the red fluorescence, that reports growth of the fluorescently tagged PS(KO) (Fig. S13B). For high ITC concentrations (30 and 60 µg/mL), the total OD_600_ clearly depends on SaxA, since it is roughly twice for the PS:K coculture as for PSKO:K (Fig. S13A). However, red fluorescence, indicating the pathogen, is similar between PS and PSKO (Fig. S13B), indicating that the increase in OD_600_ is likely mostly due to the rescue of K, as predicted by the model.

### Commensal ITC sensitivity influences the SaxA-mediated biocontrol effect on PS

To understand how the interplay between commensal rescue and nutrient competition functions for different commensals, we also generated model predictions for the least ITC-sensitive commensal strain, *Stenotrophomonas* sp. E. Because E is only weakly ITC-sensitive (Fig. 1B), its rescue by PS is predicted to be mostly negligible. Thus, although nutrient competition with E suppresses the growth of PS, the model predicts that SaxA-dependent biocontrol mostly does not occur because E does not need to be rescued to achieve substantial growth rates (Fig. S14 and S15). We experimentally tested the model predictions using PS(KO):E coculture at different ratios and ITC concentrations, finding good agreement between the model predictions and the experimental data (Fig. S16). Thus, our analysis using rescue, suppression, and biocontrol indices shows that the “virulence” factor SaxA can indirectly benefit ITC-sensitive commensals, depending on their ITC sensitivity.

### Pathogen and commensal traits shape the outcome of SaxA-mediated ITC degradation

**Fig 10:**
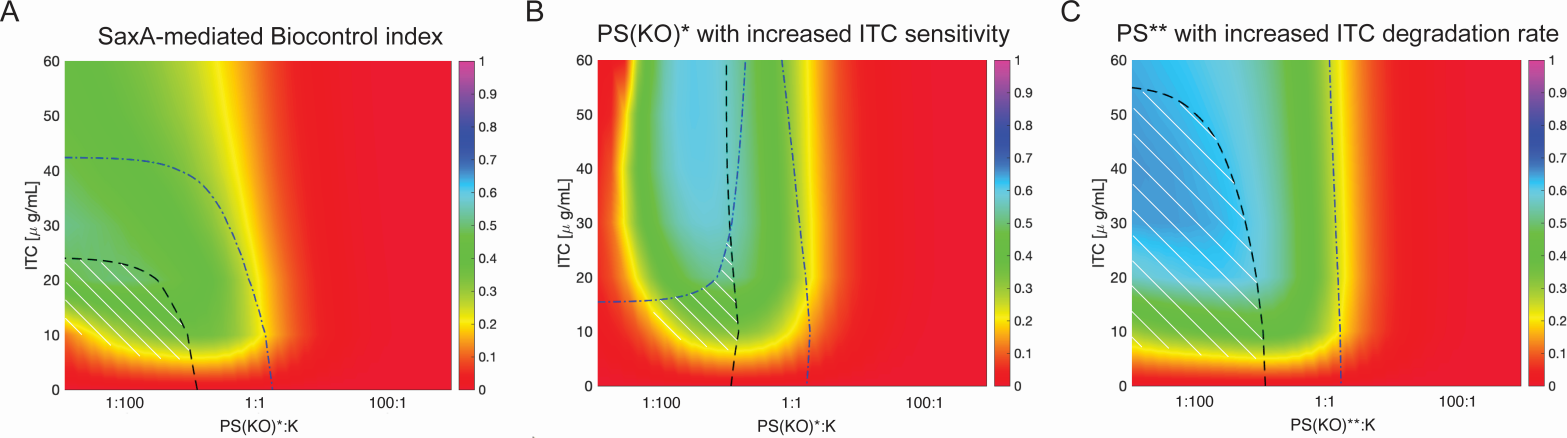
Biocontrol index maps predicted for a hypothetical pathogen PS* and/or PS** compared to PS. (**A**) SaxA-dependent biocontrol for natural PS(KO) and K, as shown in Fig. 8. (**B**) SaxA-mediated biocontrol index for hypothetical pathogen PS* with 10-fold higher ITC sensitivity (10-fold smaller value of θ in the mathematical model; equation 8 in Text S2). (**C**) SaxA-mediated biocontrol index for PS** with a 10-fold increased ITC degradation rate (10-fold larger value of λ in the mathematical model; equation 12 of Text S2). Red regions depict potential negative effects for the plant host (no rescue of commensals, no suppression of the pathogen); green/blue regions show potential positive effects, which are mediated by SaxA (rescue of commensals, suppression of the pathogen). The black dashed and blue dot-dashed lines represent where the maximal OD_600_ of modified PS and K are at 0.06. The white hatched area defines the region where the SaxA-mediated biocontrol is maximal and satisfies the condition where max OD_600_(PS) < 0.06 and max OD_600_(K) > 0.06.

To assess how trait variation influences the virulence role of SaxA, we modified the model parameters *in silico* to predict the outcome of SaxA-mediated ITC degradation in different systems (Fig. 10; Fig. S17 to S25). PS displays strong ITC-degrading capacity and low ITC sensitivity (Table S4). We modeled a variant, PS(KO)*, with 10-fold higher ITC sensitivity (θ is 10-fold lower, equation 1, Fig. 1B). Although K suppresses PS* more effectively than PS at high ITC concentrations, the SaxA-dependent biocontrol region is predicted to be narrower (Fig. 10A and B)because rescue of K is impaired at low PS*:K ratios, since higher PS* abundance is required for ITC degradation (Fig. S17B vs A). Conversely, increasing PS resistance by lowering θ 10-fold does not change the patterns of rescue, suppression, or biocontrol (Fig. S18; Fig. 8), suggesting a limit to the benefits of ITC resistance for PS and K.

We next examined how the ITC-degradation capacity of the pathogen (λ; equation 3, Fig. 1B) changes the outcome. A simulated variant PS** with 10-fold higher λ (Fig. 10C; Fig. S17C) expands the SaxA-dependent biocontrol region, consistent with higher pathogen-commensal competition. Conversely, lowering λ contracts the SaxA-dependent biocontrol region (Fig. S19).

Simulated cocultures with a commensal K* that is less sensitive to ITC resembled our predictions for strain E, showing minimal rescue benefit (Fig. S25), while a more ITC-sensitive K* was predicted to suppress PS less efficiently (Fig. S24). Thus, an optimal balance of ITC sensitivity and nutrient competition appears to be critical for the biocontrol efficacy of the commensal. Finally, modifying the intrinsic growth rates of both the pathogen and commensal (μ; equation 1, Fig. 1B) also altered the outcome. Increasing commensal growth or decreasing pathogen growth (twofold changes) expanded the biocontrol region (Fig. S21 and S22), whereas the opposite shifts contracted it (Fig. S20 and S23). Together, these simulations provide quantitative predictions for the conditions that favor SaxA-mediated commensal rescue and pathogen suppression, highlighting potential strategies for biocontrol in bacterial leaf communities.

## DISCUSSION

On the plant leaf, the growth and virulence of opportunistic pathogens like *Pseudomonas viridiflava* depend on both the leaf chemical environment and interactions with other microbes and, reciprocally, commensal colonization can depend on the pathogen. Here we show that the toxicity of plant specialized metabolites such as GLS-derived ITCs shapes the outcome of these pathogen-commensal interactions. SaxA-mediated degradation of 4MSOB-ITC benefits both *P. viridiflava* and diverse ITC-susceptible commensals, so SaxA acts as a public good that rescues commensals from ITC toxicity. The rescued commensals can in turn limit pathogen growth through resource competition. The balance of this tradeoff depends on the traits of the competing microbes, their relative abundances and the ITC level. Translating our results to the plant context, the fact that increasing toxin levels might reduce pathogen growth and virulence, while at the same time having negative effects on plant health by reducing competition with pathogen-suppressing commensals, indicates that plant defense strategies and pathogen virulence factors should be reevaluated from a broader perspective.

### Detoxification facilitates commensals, which in turn constrain the pathogen

GLS-derived breakdown products such as 4MSOB-ITC are important barriers to leaf colonization by non-adapted pathogens and commensals (3, 18, 36). We previously showed that *P. viridiflava* 3D9 (here: PS) can rescue the commensal *Plantibacter* sp. 2H11-2 (here: G) in an ITC-concentration-dependent manner (32). This is similar to other known microbial public good effects such as antibiotic degradation (37–39). Here we extended this system with four additional commensals that differ in 4MSOB-ITC sensitivity. For most of them, growth in coculture and in a synthetic community likewise depended on ITC degradation by PS, while PS growth was in turn limited by nutrient competition with the commensal. A simple dynamical model accounting only for growth, nutrient depletion, ITC suppression and cell death reproduced the coculture dynamics quantitatively in most cases, indicating that these few interactions dominate *in vitro.*

Antimicrobial compounds are widespread in many environments. A comparable public-good effect occurs in toxic metalworking fluids, where detoxification facilitates the growth of co-cultured bacteria, but competition increases and facilitation declines as nutrients become more abundant (40). More generally, in resource-limited communities containing antimicrobials, detoxification as a public good is expected to give rise to competition, a dynamic likely relevant to leaf surfaces and apoplasts, which combine diverse antimicrobial metabolites with low nutrient levels. We note that our model does not exclude an additional role for the breakdown product 4MSOB-amine as a nitrogen source under the nutrient-limited conditions of the leaf.

### The tradeoff between SaxA as virulence factor and public good depends on ITC, pathogen, and commensal traits

We next used the calibrated model to explore how microbial traits, such as ITC degradation rate, growth rate and ITC sensitivity, shape SaxA-mediated interactions. Varying these parameters *in silico* showed that the outcome is strongly context dependent. We term the sequence of detoxification followed by competitive suppression of PS “SaxA-mediated biocontrol” since pathogen suppression through nutrient competition is itself an established biocontrol principle (41). The model predicts that biocontrol strengthens when the pathogen degrades ITC faster and when PS is outnumbered by commensals; it and weakens when the pathogen is more ITC-sensitive. Such trait variation is plausible in nature: SaxA enzymes differ in catalytic activity depending on phylogenetic origin (19), SaxF-like efflux pumps vary in their protective efficiency (42), and both degradation and sensitivity depend on the chemically diverse ITCs of different Brassicaceae plants (19, 42, 43). The interaction outcome therefore depends on the traits of all three parties—plant host, pathogen, and commensal.

### ITC detoxification might be especially important for commensals when high ITC levels are released from a plant leaf

ITC degradation by PS removes the function of these plant defence metabolites, which could promote disease by impairing the plant’s control over opportunistic pathogens and its microbiome (44); yet our results show it can instead drive SaxA-mediated biocontrol. Our model predicts this biocontrol is favored when the ITC concentration exceeds a commensal-specific threshold and the pathogen:commensal ratio is low. We might find such conditions at the onset of disease in a leaf. *Pseudomonas viridiflava* is a common, low-abundant colonizer of healthy *A thaliana* leaves (13), and ITC levels rise rapidly in wounded tissue, e.g. after herbivory (15). Our model predicts that under such conditions ITC-sensitive commensals like *Janthinobacterium* sp. K would benefit indirectly from SaxA and could help suppress the pathogen. Up to now the ITC concentration to which individual commensal cells are exposed during such an attack has not been quantified. One study measured the bulk apoplastic concentration of 4MSOB-ITC of non-infected *A. thaliana* leaves (~42 µM = 7.4 µg/mL) (45). These concentrations are so low that according to our model ITC-sensitive commensals like K would not benefit from SaxA but could nevertheless suppress PS. Another study measured a five times increase of 4MSOB-ITC in the whole leaf sample when the pathogenic fungus Sclerotinia sclerotiorum infected and decayed *A. thaliana* leaf tissue compared to the healthy, intact leaf (20). This would correspond to at least an average of 37 µg/mL in the apoplastic fluid of infected leaves. However, without spatial information on ITC concentrations in leaves, it is difficult to estimate the exposure of individual bacterial cells to this plant toxin. Some bacterial cells might well be exposed to higher ITC concentrations locally where they might benefit from SaxA-mediated detoxification. In order to assess the relevance of our predictions *in planta*, further experiments and spatially resolved ITC data in leaves are necessary.

### Commensal rescue might indirectly benefit an opportunistic pathogen

As a public good, SaxA non-selectively rescues any sensitive commensal close enough to benefit from its ITC degradation. Enriching diverse commensals could benefit the host, because their varied resource niches let them compete with opportunistic pathogens—observed in both root and leaf microbiomes (8, 9)—and because they can suppress pathogenicity by competing for virulence-inducing nutrients such as fructose or certain amino acids (46, 47). At any rate, our results suggest that SaxA-dependent biocontrol can occur under a variety of conditions, suggesting it could be rather common. This raises the question of why PS would rely on a public-good mechanism at all, given that private resistance such as ITC efflux pumps would protect it without enriching competitors. In the resource-poor leaf environment (11, 48), one possibility is that rescued commensals reciprocate, supplying nutrients or vitamins by cross-feeding (49, 50) or helping pathogens access host resources (3), allowing opportunistic pathogens to occupy new niches and integrate into a healthy microbiome. Consistent with a role beyond virulence, ITC degradation, probably via SaxA-like enzymes, was surprisingly prevalent even among our commensal leaf colonizers. We therefore propose that SaxA is a “dual-use trait” (51) echoing the idea that virulence traits of human pathogens can arise in environmental niches because they also confer advantages outside disease (52). Such context-dependent functions, conferring virulence in one setting but benefiting commensal competitors in another, may also shape evolutionary trajectories: a pathogen under pressure to cause disease should adapt differently than one embedded in a healthy microbiome, which could help explain the stable coexistence of distinct *P. viridiflava* lineages in healthy *A. thaliana* leaves (13). Resolving the roles of SaxA in the leaf microbiome will require further work, but our results already argue for considering virulence traits in their condition- and community-dependent context.

### Model scope and limitations

Mathematical modeling was central to this work: a simple dynamical model calibrated on monoculture growth data quantitatively reproduced coculture interactions and predicted untested scenarios, raising ecological questions for plant–pathogen–commensal systems. The model is, of course, a simplification. It represents only one or two sequentially consumed nutrients, whereas leaves allow nutrient co-utilization (53, 54, 55), and secretion of secondary metabolites (56); in addition, the rich, undefined R2A medium used here generates complex utilization patterns. We deliberately kept the model minimal to avoid overfitting and preserve interpretability; more highly parameterized versions with up to three carbon sources and varied utilization orders did not improve the fits. The present parameterization is specific to our strains, and applying it to new community compositions would require *in vitro* screening to re-derive growth parameters and capture possible byproduct cross-feeding. The framework itself, however, should be robust: simple consumer-resource models, often without diauxic shifts, can predict the assembly, steady states and dynamics of complex microbial communities across resource conditions (57–59). ITC degradation and toxicity were modeled phenomenologically, without explicit molecular mechanisms, yet this sufficed to describe the *in vitro* system. Our analysis mainly focused on single-species and pairwise cultures, whereas the study of complex microbial communities, especially in a biofilm context such as on leaves, may involve higher-order interactions (60, 61). In addition, we did not include pH or temperature shifts (62, 63), secreted secondary metabolites (64), species invasion or migration (65), and evolutionary dynamics (66). Bacteria on leaves are also spatially non-uniformly distributed (67), and capturing this would require a spatially explicit modeling framework, which lies beyond the scope of the present work (68, 69). Within these bounds, a minimal phenomenological model with few key factors explained pairwise coculture interactions and generated testable, ecologically relevant predictions.

## Data Availability

All custom code used for data analysis, growth curve fitting, and simulation of the model scenarios presented in the main text and supplemental information is publicly available at https://sourceforge.net/projects/pathogen-commensal-interaction/files/Bacterial_degradation.zip/download. The repository includes scripts, documentation, and example workflows to reproduce the reported results. Amplicon sequencing data generated and analyzed in this study are available at https://www.ncbi.nlm.nih.gov/bioproject/1456038. All other data supporting the findings of this study are available from the corresponding author upon reasonable request.

## SUPPLEMENTAL MATERIAL

Text S1 msystems.00064-26-s0001.pdf Effect of ITC on yield coefficient.

Text S2 msystems.00064-26-s0002.pdf Extension of model to two nutrients.

Text S3 msystems.00064-26-s0003.pdf Development of rescue, suppression, and biocontrol indices.

Supplemental Material msystems.00064-26-s0004.docx Fig. S1 to S25; Tables S1 to S4.

ASM does not own the copyrights to Supplemental Material that may be linked to, or accessed through, an article. The authors have granted ASM a non-exclusive, world-wide license to publish the Supplemental Material files. Please contact the corresponding author directly for reuse.
