# Supplementary material for: Bacterial degradation of a plant toxin and nutrient competition with commensals trade off to constrain pathogen growth
Source: mSystems. 2026 Jun 26;11(7):e00064-26. doi: 10.1128/msystems.00064-26 (PMC13386982; doi:10.1128/msystems.00064-26)
Supplement: Text S1 — Effect of ITC on yield coefficient. [file msystems.00064-26-s0001.pdf]

ITC has the effect of modifying the maximal OD and thus the yield coefficient

Bacterial growth is usually described by the assimilation of substrate S that is transformed into biomass B via an ordinary differential equation system

$$\frac{d[B(t)]}{dt} = \mu\frac{[S(t)]}{[S(t)] + \theta_S}[B(t)] \tag{1}$$

$$\frac{d[S(t)]}{dt} = -Y\frac{d[B(t)]}{dt} \tag{2}$$

where Y represent the yield that converts the units of the substrate (e.g. g/L) into biomass units (e.g. $a.u.$ or cell/volume) and expresses how much nutrient amount is necessary to make a unit of biomass. Such equations obey to the mass conservation principle, so that their net sum is zero

$$\frac{d[B(t)]}{dt} + \frac{1}{Y}\frac{d[S(t)]}{dt} = 0 \tag{3}$$

expressing the fact that no substrate is lost in the assimilation into biomass. In the monocultures, we observed that the maximal OD is reduced as a function of the ITC concentration and we hypothesize that this leads to an impaired assimilation of the substrate. Therefore, we modified the ODE system including a loss term (Fig. 1), dependent on the ITC concentration, expressed as the proportion α (a number between 0 and 1) of substrate that is transformed into biomass. The ratio $1 - \alpha$ is instead lost into L. Accounting for the lost substrate L, the ODE system becomes

$$\frac{d[B(t)]}{dt} = -\frac{\alpha}{Y}\frac{d[S(t)]}{dt} \tag{4}$$

$$\frac{d[L(t)]}{dt} = -\frac{1-\alpha}{Y}\frac{d[S(t)]}{dt} \tag{5}$$

$$\frac{d[S(t)]}{dt} = -Y\frac{d[B(t)]}{dt} - Y\frac{d[L(t)]}{dt}. \tag{6}$$

Neglecting the loss term as we are not interested in its dynamics in our system and imposing the Monod equation for the substrate assimilation, we obtain

$$\frac{d[B(t)]}{dt} = \alpha\mu\frac{[S(t)]}{[S(t)] + \theta_S}[B(t)] \tag{7}$$

$$\frac{d[S(t)]}{dt} = -Y\mu\frac{[S(t)]}{[S(t)] + \theta_S}[B(t)]. \tag{8}$$

From Equation 4, we notice the the system converts substrate into biomass via an effective yield

$$y = \frac{Y}{\alpha} \tag{9}$$

that depends on α. We suppose then the ratio of nutrient assimilated into biomass α is a saturable function of the ITC concentration

$$\alpha(ITC) = \frac{\theta}{[ITC(t)] + \theta}, \tag{10}$$

so that ITC has the effective results of altering the yield y in Equation 9

$$y = Y\frac{[ITC(t)] + \theta}{\theta} \tag{11}$$

that reduces to the normal yield in the absence of ITC and is smaller than Y in the presence of ITC, expressing the diverting of the nutrient from the biomass pathway. We can finally rewrite the whole system by inserting the definition of α in Equations 7 and 8

$$\frac{d[B(t)]}{dt} = \mu\frac{[S(t)]}{[S(t)] + \theta_S}\frac{\theta}{[ITC(t)] + \theta}[B(t)] \tag{12}$$

$$\frac{d[S(t)]}{dt} = -\mu Y\frac{[S(t)]}{[S(t)] + \theta_S}[B(t)]. \tag{13}$$

Including the possibility to degrade ITC with a rate λ by PS and a death rate term δ lead to the ODE of the main text

$$\frac{d[B(t)]}{dt} = \mu\frac{[S(t)]}{[S(t)] + \theta_S}\frac{\theta}{[ITC(t)] + \theta}[B(t)] - \delta[B(t)] \tag{14}$$

$$\frac{d[S(t)]}{dt} = -\mu Y\frac{[S(t)]}{[S(t)] + \theta_S}[B(t)] \tag{15}$$

$$\frac{d[ITC(t)]}{dt} = -\lambda\frac{[ITC(t)]}{[ITC(t)] + \theta_{ITC}}[B(t)],\ 0. \tag{16}$$

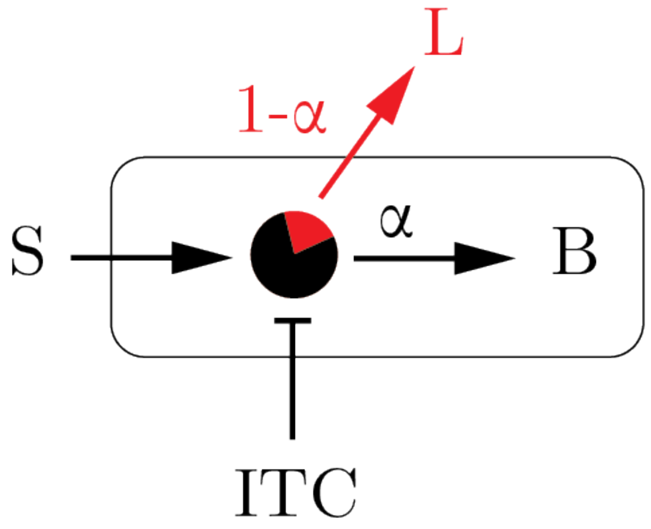

Figure 1: The substrate S is assimilated by the cell and a fraction α is transformed into biomass B, while the rest $1-\alpha$ is lost as L. ITC has the effect of increasing the coefficient α suppressing the biomass formation.
