# Supplementary material for: Bacterial degradation of a plant toxin and nutrient competition with commensals trade off to constrain pathogen growth
Source: mSystems. 2026 Jun 26;11(7):e00064-26. doi: 10.1128/msystems.00064-26 (PMC13386982; doi:10.1128/msystems.00064-26)
Supplement: Text S2 — Extension of model to two nutrients. [file msystems.00064-26-s0002.pdf]

Extension of the model to two nutrients

The dynamics of the OD of the pathogens (PS, PSKO) show a change in the slope between 10 and 20 hours depending on the ITC concentration (Fig. 4 of the main text), probably indicating a switch in the nutrient utilization. To account for it, we modified our original model

$$\frac{d[B(t)]}{dt} = \mu\frac{[S(t)]}{[S(t)] + \theta_S}\frac{\theta}{[ITC(t)] + \theta}[B(t)] - \delta[B(t)] \tag{1}$$

$$\frac{d[S(t)]}{dt} = -\mu Y\frac{[S(t)]}{[S(t)] + \theta_S}[B(t)] \tag{2}$$

$$\frac{d[ITC(t)]}{dt} = -\lambda\frac{[ITC(t)]}{[ITC(t)] + \theta_{ITC}}[B(t)],\ 0 \tag{3}$$

including a diauxic shift from the first nutrient S_1 to a second nutrient S_2

$$\frac{d[B(t)]}{dt} = \left(\mu_1\frac{[S_1(t)]}{[S_1(t)] + \theta_{S1}} + \mu_2\frac{[S_2(t)]}{[S_2(t)] + \theta_{S2}}\frac{\theta_N}{[S_1(t)] + \theta_N}\right)\frac{\theta}{[ITC(t)] + \theta}[B(t)] - \delta[B(t)] \tag{4}$$

$$\frac{d[S_1(t)]}{dt} = -\mu_1 Y_1\frac{[S_1(t)]}{[S_1(t)] + \theta_{S1}}[B(t)] \tag{5}$$

$$\frac{d[S_2(t)]}{dt} = -\mu_2 Y_2\frac{[S_2(t)]}{[S_2(t)] + \theta_{S2}}\frac{\theta_N}{[S_1(t)] + \theta_N}[B(t)] \tag{6}$$

$$\frac{d[ITC(t)]}{dt} = -\lambda\frac{[ITC(t)]}{[ITC(t)] + \theta_{ITC}}[B(t)],\ 0 \tag{7}$$

where μ_i, θ_{Si}, and Y_i are the maximal growth rate,the Monod constant, and the yield coefficient of the nutrient i, and θ_N the diauxic suppression constant indicating the concentration at which the first nutrient induces half maximal suppression on the assimilation of the second carbon source.

Extension of the model to pairwise culture

While growing together in pairwise culture, pathogen and commensal co-utilize the substrate. For simplicity, we suppose that they share the substrate S_1. If B and C represent the biomass of the pathogen and commensal, respectively, the system of ODE describing our

model becomes

$$\frac{d[B(t)]}{dt} = \left(\mu_1 \frac{[S_1(t)]}{[S_1(t)] + \theta_{S1}} + \mu_2 \frac{[S_2(t)]}{[S_2(t)] + \theta_{S2}} \frac{\theta_N}{[S_1(t)] + \theta_N}\right) \frac{\theta}{[ITC(t)] + \theta}[B(t)] - \delta[B(t)] \tag{8}$$

$$\frac{d[C(t)]}{dt} = \mu_C \frac{[S_1(t)]}{[S_1(t)] + \theta_{SC}} \frac{\theta_C}{[ITC(t)] + \theta_C}[C(t)] - \delta[C(t)] \tag{9}$$

$$\frac{d[S_1(t)]}{dt} = -\mu_1 Y_1 \frac{[S_1(t)]}{[S_1(t)] + \theta_{S1}}[B(t)] - \mu_C Y_C \frac{[S_1(t)]}{[S_1(t)] + \theta_{SC}}[C(t)] \tag{10}$$

$$\frac{d[S_2(t)]}{dt} = -\mu_2 Y_2 \frac{[S_2(t)]}{[S_2(t)] + \theta_{S2}} \frac{\theta_N}{[S_1(t)] + \theta_N}[B(t)] \tag{11}$$

$$\frac{d[ITC(t)]}{dt} = -\lambda \frac{[ITC(t)]}{[ITC(t)] + \theta_{ITC}}[B(t)],\ 0 \tag{12}$$

where we rename the set of parameters with subscript $_C$ for the substrate consumption by the commensal.

Including a Hill effect of ITC repression

We noticed that in some cases, such as for the commensal K and G, the ITC repression does not follow the function

$$\frac{\theta}{[ITC(t)] + \theta} \tag{13}$$

but rather a sigmoidal function represented by the introduction of a Hill coefficient m

$$\frac{\theta^m}{[ITC(t)]^m + \theta^m} \tag{14}$$

that we use in our model and for fitting the single culture OD dynamics.
