# Supplementary material for: Bacterial degradation of a plant toxin and nutrient competition with commensals trade off to constrain pathogen growth
Source: mSystems. 2026 Jun 26;11(7):e00064-26. doi: 10.1128/msystems.00064-26 (PMC13386982; doi:10.1128/msystems.00064-26)
Supplement: Text S3 — Development of rescue, suppression, and biocontrol indices. [file msystems.00064-26-s0003.pdf]

Development of the rescue, suppression, and biocontrol indexes

In the main text, we defined the rescue, suppression, and SaxA-dependent biocontrol indexes as a function of the maximal OD attained in different conditions of ITC concentrations and pathogen:commensal initial mixing ratio as

$$\text{Rescue index} = 1 - \frac{\text{Max OD}(K \text{ with PSKO})}{\text{Max OD}(K \text{ with PS})} \tag{1}$$

$$\text{Suppression index} = 1 - \frac{\text{Max OD}(PS \text{ with K})}{\text{Max OD}(PS)} \tag{2}$$

$$\text{Biocontrol index} = \text{Rescue index} \cdot \text{Suppression index} \tag{3}$$

that, for the cases discussed in the main text, are numbers ranging from 0 to 1. The rescue index quantifies the conditions where the presence of a SaxA-degrader PS enhance the maximal biomass produced by the commensal compared to the case in the absence of degrader (such as PSKO). The suppression index quantifies the conditions where the maximal biomass attained by the pathogen ITC-degrader PS is repressed by growing in the presence of commensal K, due to nutrient competition. Finally, the SaxA-dependent biocontrol index combines the two previous indexes indicating the conditions when the plant could benefit of a control of the pathogen PS number by the presence of the commensal exploiting the trade-off between the commensal rescue due to sharing the effect of SaxA on ITC degradation and the PS repression because of the nutrient competition with the rescued commensal. To understand better the dynamics of the biomass in different scenario of ITC concentrations and pathogen:commensal initial ratios, we refer to Fig. 12 in the supplementary text, replotted here as upper panel of Fig. 1.

The central panel of Fig. 1A illustrates the case where PS (or PSKO) and K are introduced at equal initial concentrations (PS(KO):K = 1:1). In this condition, the maximal optical density (OD_{600}) of K co-cultured with PSKO declines from 0.08 (blue dashed line) to below 0.005 when exposed to ITC concentrations exceeding 15 μg/mL (red dashed line, lower panel). By contrast, PS, thanks to its ability to degrade ITC, partially rescues K's growth; at 15 μg/mL ITC, the OD_{600} reaches approximately 0.05 (upper panel, red dashed line). Due to the high ITC-degrading capacity of PS, even a small number of PS cells is sufficient to effectively eliminate ITC and restore K proliferation, as demonstrated in Fig. 1A where the initial PS concentration is reduced by two orders of magnitude relative to K (PS(KO):K = 1:100). In this scenario, the recovered growth of K leads to increased nutrient competition, which in turn suppresses PS proliferation. For instance, at 15 μg/mL ITC, the maximal OD_{600} of PSKO is 0.08 (solid red line), whereas that of PS drops to 0.06 (lower panel, first column). The impact of nutrient competition becomes even more apparent when comparing PS growth in the presence versus absence of K (Fig. 1B, first rows of panel A vs. panel B). For example, at a PS:K ratio of 1:100 and 60 μg/mL ITC, PS growth decreases from 0.19 to 0.14 (black solid line),

and in the absence of ITC, from 0.19 to 0.05 (blue solid line). Conversely, when the initial PS abundance greatly exceeds that of K (PS(KO):K = 100:1, Fig. 1, rightmost columns), PS monopolizes nutrient resources, resulting in negligible K growth, even when rescued by ITC-degrading PS (dashed lines, upper panel).

We used the dynamics of the biomass for different conditions as presented in Fig. 1 to extract the maximal biomass values (supposed proportional to OD), as summarised in the heat maps of Suppl. Fig. 13. Then, the maximal OD served to compute the values of the indexes for several conditions and summarised in the corresponding heat maps.

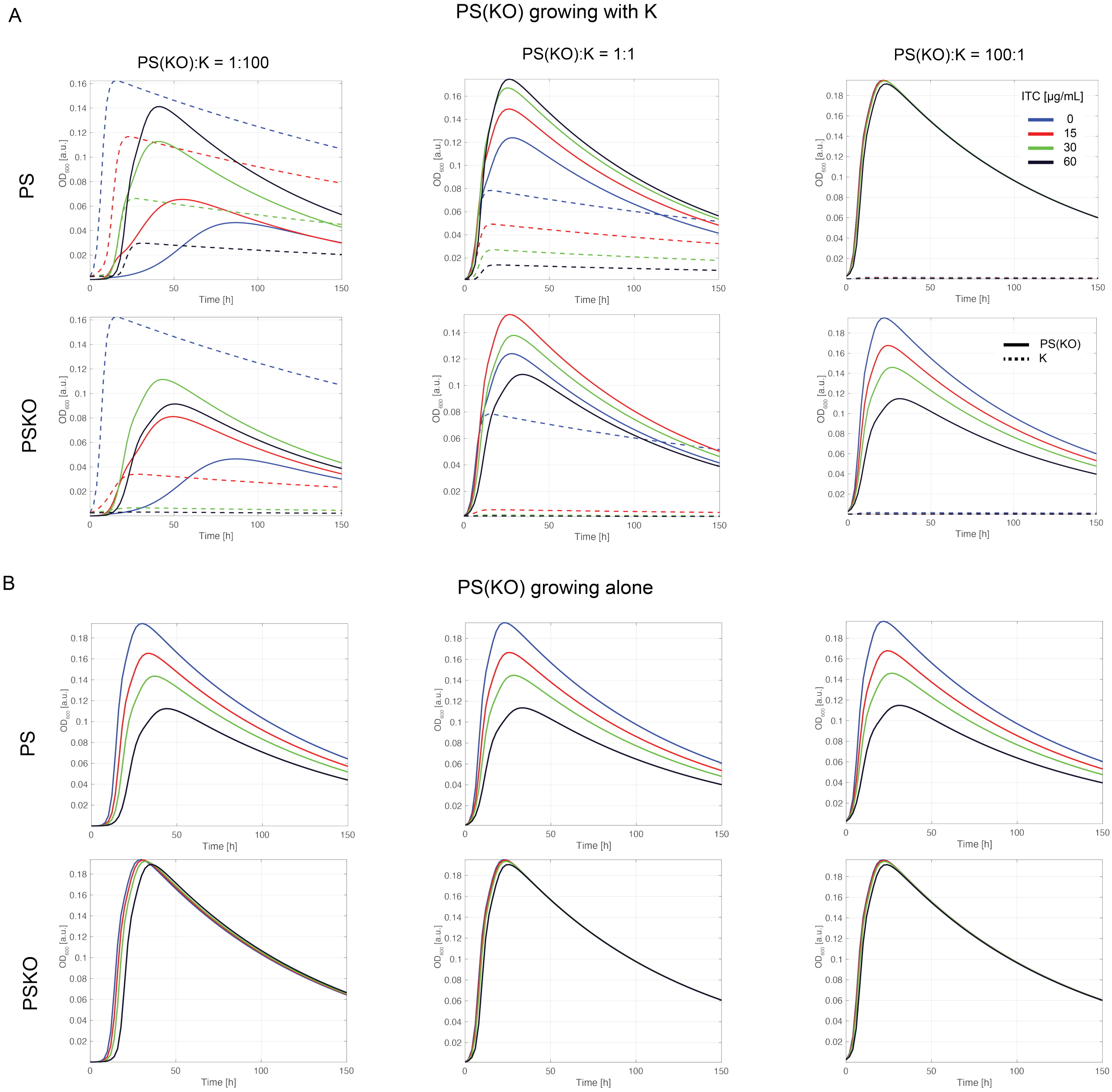

Figure 1: OD$_{600}$ dynamics of pathogens PS and PSKO growing in the absence or presence of commensal K for several initial ratio pathogen:K. (A) OD dynamics of either PS or PSKO (solid lines, top and bottom row, respectively) and K (dashed lines) for several ITC concentrations and three initial pathogen:K ratio (1:100, 1:1, 100:1, first, second and third column, respectively). Panel (B) shows the dynamics of the monocultures of either PS or PSKO, in the absence of commensal K, with the same initial values as in A.
